# Light responsive nucleic acid for biomedical application

**DOI:** 10.1002/EXP.20210099

**Published:** 2022-04-06

**Authors:** Liwei Chen, Yanfei Liu, Weisheng Guo, Zhenbao Liu

**Affiliations:** ^1^ Department of Pharmaceutical Engineering College of Chemistry and Chemical Engineering Central South University Changsha Hunan Province P. R. China; ^2^ Department of Pharmaceutics Xiangya School of Pharmaceutical Sciences Central South University Changsha Hunan Province P. R. China; ^3^ Department of Minimally Invasive Interventional Radiology Guangdong Provincial Key Laboratory of Molecular Target & Clinical Pharmacology, the NMPA and State Key Laboratory of Respiratory Disease School of Pharmaceutical Sciences & The Second Affiliated Hospital Guangzhou Medical University Guangzhou Guangdong Province P. R. China; ^4^ Molecular Imaging Research Center of Central South University Changsha Hunan Province P. R. China

**Keywords:** biomedical application, photocleavage, photocrosslinking, photoisomerization, photoresponsive nucleic acid

## Abstract

Nucleic acids are widely used in biomedical applications because of their programmability and biocompatibility. The light responsive nucleic acids have attracted wide attention due to their remote control and high spatiotemporal resolution. In this review, we summarized the latest developments in biomedicine of light responsive molecules. The molecules which confer light responsive properties to nucleic acids were summarized. The binding sites of molecules to nucleic acids, the induced structural changes, and functional regulation of nucleic acids were reviewed. Then, the biomedical applications of light responsive nucleic acids were listed, such as drug delivery, biosensing, optogenetics, gene editing, etc. Finally, the challenges were discussed and possible future directions of light‐responsive nucleic acids were proposed.

## Graphical abstract

Light‐responsive nucleic acids have attracted much attention in the biomedical field due to their properties of remote control and high spatiotemporal resolution. This review summarizes recent research progress in light‐responsive nucleic acid systems. The challenges in this field are discussed and future prospects are prospected. This review will provide inspiration for the rational design of light‐responsive nucleic acid systems.

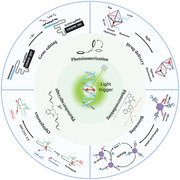

## 1 INTRODUCTION

With the programmability and predictability based on sequence‐specific hybridization,^[^ ^1^ ^]^ the nucleic acid has become an outstanding functional material for rationally designing nano‐scale molecular machines with multiple functions,^[^ ^2^ ^]^ including recognition, replication, transport, and selective catalytic activities, which makes nucleic acid attractive to scientists to explore its applications in various fields.^[^ ^3^ ^]^ Nucleic acids have been used as building blocks or templates for the construction of highly ordered biological materials. The highly predictable, specific binding and folding capabilities of nucleic acids make it possible to design complex two‐dimensional and three‐dimensional soft materials.^[^ ^4^ ^]^ The molecules recognition characteristics and polymorphisms of nucleic acids make it an ideal scaffold for designing complex supramolecular structures, which can control their composition, structures, and functions.^[^ ^5^ ^]^ Furthermore, nucleic acids have been used to design systems in response to specific stimuli.^[^ ^4^, ^6^ ^]^

Stimuli‐responsive regulation of biomolecule‐ligand complexes is a powerful tool for controlling biological functions, and it is also a potential source of new tools in biochemistry, nanotechnology, materials science, pharmacology, and medicine.^[^ ^7^ ^]^ In the past few decades, stimuli‐driven nanomaterials have attracted widespread attention and have played a pivotal role in many fields, including therapeutic diagnostics research,^[^ ^8^ ^]^ biochemical sensing,^[^ ^9^ ^]^ self‐healing materials,^[^ ^10^ ^]^ and energy study.^[^ ^11^ ^]^ These stimuli‐driven nano‐systems can sensitively change their physical or chemical properties, and realize on‐demand “OFF/ON” switching when internal/external stimuli are applied.^[^ ^12^ ^]^ Scientists have invested much effort in the field of stimulus responsiveness of nucleic acid structures and have developed various stimuli, such as pH,^[^ ^13^ ^]^ heat,^[^ ^14^ ^]^ small molecules,^[^ ^4^ ^]^ enzymes,^[^ ^15^ ^]^ metal ions,^[^ ^16^ ^]^ and specific nucleic acid sequences,^[^ ^5^ ^]^ for the triggering of these pre‐programmed nucleic acid nanostructures to change their structure. For example, in biomedical applications, Sun et al.^[^ ^17^ ^]^ designed a pH‐responsive DNA nano‐drug delivery system. Under low pH conditions of tumor cells, the C‐rich sequence on the DNA nanocarrier was protonated and formed an i‐motif structure. Then, the GC double‐strands were dehybridized, thereby releasing the doxorubicin (DOX) embedded in the DNA duplex. Yu et al.^[^ ^18^ ^]^ reported a DNA‐gated nanocarrier that could controllably release drugs at different temperatures without any external stimulation. When the temperature was raised, the weak electrostatic interaction between the mesoporous silica and DNA was destroyed, causing the valve to be in an “open” state, allowing the release of the cargo molecules from the nanocarrier. In Khalil's group,^[^ ^19^ ^]^ they demonstrated a non‐chemotherapeutic drug delivery system that uses DNA nanostructures to co‐deliver antimicroRNA‐21 (antimiR‐21) and KLA peptides for breast cancer treatment. After being internalized into tumor cells, the high ATP concentration caused DNA nanostructures to break down and restore antimiR‐21 function. It could lead to effective inhibition of tumor proliferation in vitro and in vivo, and induce synergistic anti‐cancer activity. Zhang and colleagues^[^ ^20^ ^]^ integrated DNA templated quantum dots (QDs) into DNA hydrogel networks through self‐assembly and designed an enzyme‐responsive quantum dot DNA hydrogels (QDHs). QDHs are stable in a series of physiologically relevant temperatures and pH ranges, but once entered into cells through nuclease digestion, they will be degraded, releasing the DNA‐binding drug DOX. Park et al.^[^ ^16^ ^]^ developed a strategy to regulate DNA polymerase activity through the interaction between metal ions and DNA bases. When Hg^2+^ was added, the bridging of Hg^2+^ and TT pairs would change the secondary structure of the DNA aptamer and affect its ability to inhibit DNA polymerase. With the addition of strong metal ion binder cysteine, the ability of the DNA aptamer to inhibit DNA polymerase was restored. The specific nucleic acid sequence responsive system induces the reconfiguration of DNA nano‐components through the foothold‐mediated strand displacement reaction, and is often used in biological imaging. Li et al.^[^ ^21^ ^]^ designed a nucleic acid sequence‐responsive probe to realize simple and visual imaging of microRNA (miRNA) at the femtomolar level through a strand replacement cascade mediated by an enzyme‐free foothold. These stimuli can effectively trigger the nucleic acid systems to release drugs, biological imaging, or affect physiological activities. However, it can be seen from the above examples that a large number of stimulating molecules will be consumed, and the waste may be rapidly generated and accumulated, affecting the performance of the entire nucleic acid system. For example, Park et al.^[^ ^16^ ^]^ used the heavy metal Hg^2+^ to trigger structural changes of DNA aptamers. This regulation leads to the accumulation of a large amount of biologically toxic Hg^2+^, especially when reversible regulation is required, which is not suitable for DNA polymerase regulation in vivo. In addition, all these reactions are triggered chemically, which do not provide real‐time control with temporal and spatial resolution. So, the external control of DNA nanostructures is important for their applications in various therapeutic and diagnostic‐related biological processes.^[^ ^22^ ^]^ Thermal stimulation may not produce waste and can be controlled externally with thermal shock, but it can only work within a limited temperature range. In addition, too high or too low temperatures, as well as thermal shock, can destroy the entire biological system.^[^ ^23^ ^]^ Therefore, the use of light provides an attractive strategy to exert control with spatiotemporal resolution.^[^ ^24^ ^]^

Light is an attractive means of controlling responsive systems.^[^ ^7^ ^]^ It offers significant advantages over chemical stimulation as it can be managed with temporal and spatial accuracy in a highly controllable and non‐invasive manner,^[^ ^25^ ^]^ and provides remote control and wavelength tunability.^[^ ^25^ ^a,^ ^26^ ^]^ Furthermore, light is compatible with most cellular components in a non‐invasive manner, and the real‐time manipulation of tissues, cells and even subcellular compartments is controllable.^[^ ^27^ ^]^ As a cleaning trigger, photoactivation has been proven to be a powerful means of external control, and has been widely used in chemical and biological systems to control the motion of molecules and regulate important processes,^[^ ^1^, ^28^ ^]^ such as performing fluorogenic photoclick labeling,^[^ ^29^ ^]^ gene delivery,^[^ ^30^ ^]^ logic gate operation,^[^ ^31^ ^]^ and enzyme activation.^[^ ^32^ ^]^ Furthermore, using light as a stimulus to manipulate the nucleic acid system can eliminate the steric hindrance at the site of action of huge DNA nanostructures. Although high‐energy light irradiation may generate heat to make the DNA system unstable, the performance of these light‐responsive platforms can be controlled by adjusting parameters such as optical power, wavelength, and exposure time.^[^ ^23^ ^]^ In addition, the low tissue penetration and phototoxicity of ultraviolet light can also be replaced by two‐photon excitation, up‐conversion luminescence, etc., or the use of near‐infrared light with better penetration.^[^ ^33^ ^]^

In order to develop DNA nano‐systems with light responsive properties, it is necessary to introduce photosensitive groups such as photocage/photocleavage molecules, photoisomerization molecules, and photocrosslinking molecules into the DNA system. They will lead to (1) hybridization and dehybridization of nucleic acid strands;^[^ ^34^ ^]^ (2) strand displacement reactions of nucleic acid sequences;^[^ ^35^ ^]^ (3) hybridization chain reactions of nucleic acid systems.^[^ ^36^ ^]^ These light‐induced nucleic acid chain reactions trigger changes in the structure of nucleic acids, which in turn lead to changes in the functional properties of nucleic acids and produce biomedical effects.^[^ ^37^ ^]^ This review introduced several photoresponsive molecules embedded in nucleic acids and their working mechanisms in response to light. The positions of light‐responsive molecules that play key roles in nucleic acids were also discussed. Some representative examples were introduced to illustrate the biomedical applications of light responsive nucleic acids.

## 2 PHOTORESPONSIVE MOLECULES

Since DNA itself is not respond to light, it is necessary to introduce some photoresponsive molecules into DNA nanostructures so that these nanostructures can respond to light.^[^ ^38^ ^]^ When exposed to light, photoresponsive molecules can affect the strength of Watson‐Crick hydrogen bonds between bases, thus affecting the complementary pairing ability of bases and changing the structure of nucleic acids.^[^ ^4^ ^]^ Here are several photoresponsive molecules used for the construction of light responsive nucleic acid systems.

### 2.1 Photocleavage/photocage molecules

**FIGURE 1:**
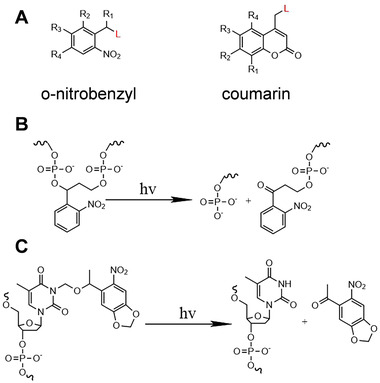
(A) Structural formula of o‐nitrobenzyl and coumarin. Schematic diagram of (B) photodecage and (C) photocleavage

Photocleavage/photocage molecules are a series of molecules that can be photocleaved, mainly including o‐nitrobenzyl groups and coumarins (Figure 1A).^[^ ^39^ ^]^ As shown in Figure 1C, the photocage compound has photocleavage groups embedded in the key position of the nucleic acid sequence, which can temporarily inactivate the biological activity of the nucleic acid. Upon exposure to light, the connection between photocage group and nucleotide will be broken, and the function of nucleic acid will be restored.^[^ ^40^ ^]^ Photocleavage molecules usually connect two or more nucleic acid sequence fragments via covalent bonds (Figure 1B). Under light irradiation, the nucleic acid sequence can be cut into small fragments by breaking the bond of the incorporated photo‐cleavable molecules, thereby restoring its functions.^[^ ^31^ ^]^ Photocleavage/photocage molecules have been widely used to regulate nucleic acids hybridization. This method can induce excellent ON/OFF behavior, form an interesting research foundation in the fields of biochemistry and materials,^[^ ^41^ ^]^ and realize the construction of useful nanostructures.^[^ ^42^ ^]^

O‐nitrobenzyl and its analogs are ideal photosensitive molecules, which are usually used as photocleavage/photocage molecules to temporarily disable nucleic acid functions. The function and biological activity of nucleic acids can be restored by light‐induced cleavage or decage of photoresponsive molecules.^[^ ^25^ ^a]^ O‐nitrobenzyl functionalized DNA/RNA structures have been developed for the spatiotemporal control of DNA and RNA structures in living cells.^[^ ^43^ ^]^

**FIGURE 2:**
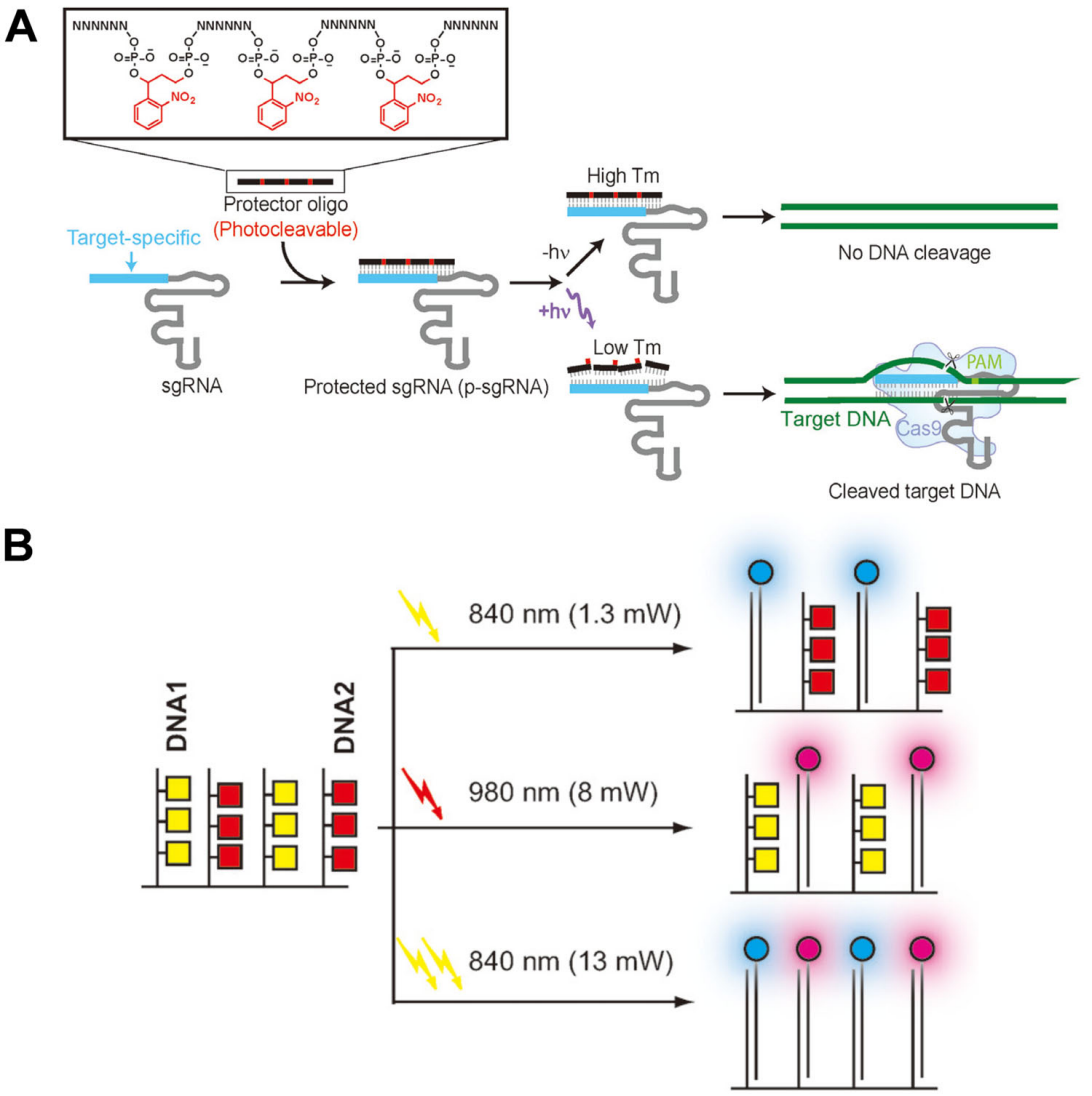
(A) The CRISPR‐plus system concept. Reproduced with permission.^[^ ^44^ ^]^ Copyright 2016, John Wiley & Sons. (B) Overview of the selective uncaging strategy. Reproduced with permission.^[^ ^48^ ^]^ Copyright 2016, John Wiley & Sons

Jain and coworkers^[^ ^44^ ^]^ designed a “CRISPR‐plus” system for the photoactivatable blockade of Cas9‐mediated DNA targeting. As shown in Figure 2A, the core structure unit of the system is the protector oligo, which is a nucleic acid sequence formed by connecting multiple nucleic acid sequence fragments with o‐nitrobenzyl groups. Before being illuminated, the protecting group hybridizes with the target recognition domain of the single chimeric guide RNA (sgRNA), making it unable to recognize and target DNA. After ultraviolet (UV) light irradiation, the connection between the photocleavage groups and the nucleic acid skeleton is broken, making the long protector oligo broken into small fragments, thus exposing the sgRNA recognition domain and restoring the ability of sgRNA to target DNA. The hybridization of sgRNA and DNA promotes its enzymatic hydrolysis and achieves the purpose of gene editing.

Despite some drawbacks, o‐nitrobenzene and its analogs may be the most popular photocleavage/photocage group among researchers in the past few years.^[^ ^39^ ^]^ For example, the release rates of o‐nitrobenzyl groups are slower than coumarin derivatives.^[^ ^39^ ^]^ In addition, o‐nitrobenzyl derivatives will generate nitrosoaldehyde after light exposure. which can react with amines. This reaction is unfavorable to surrounding proteins, thus inducing potential toxicity.^[^ ^45^ ^]^

Coumarins can be used both as a crosslinker and cleavable group, and is a good substitute for o‐nitrobenzene. Usually, there is a photocleavage ester bond between coumarin and nucleic acid skeleton.^[^ ^46^ ^]^ Under light irradiation, the connection between coumarins and the nucleic acid is broken and the function of nucleic acid is restored. Coumarins‐based light responsive nucleic acid has numerous advantages, especially the release rate of coumarin is faster than that of o‐nitrobenzene.^[^ ^39^ ^]^ Coumarins can be easily adjusted by modifying groups to adapt to their toxicity. Researchers have synthesized a lot of analogs to increase its solubility or redshift its absorption wavelengths.^[^ ^47^ ^]^

Fichte and coworker^[^ ^48^ ^]^ introduced two photocleavage groups (coumarin derivatives) into DNA strands and proved that they are suitable for three‐dimensional control of DNA hybridization. As shown in Figure 2B, there were two types of photocaging molecules introduced into two separate DNA sequences via their protected phosphoramidites. The first protected phosphoramidites were modified with [7‐(diethylamino)coumarin‐4‐yl]methyl (DEACM) on the dT residue, while the other introduced p‐dialkylaminonitrobiphenyl (ANBP) on the dG residue. Under the irradiation of light with wavelength of 840 nm and restricted power (1.3 mW), only the cages of DNA1 may be unlocked, while the cage molecules on DNA2 are intact. In contrast, when exposed to 980 nm light (8 mW), it selectively uncaged DNA2, while keeping the cage molecules on DNA1 intact. The sequential irradiation of the two different wavelengths of light or the higher power (13 mW) irradiation of 840 nm can achieve the uncaging of both DNA1 and DNA2. All these results indicate that this control method has potential in complex light control scenes.

In addition to the above‐mentioned important photosensitive molecules and their derivatives, other photocleavable molecules have been reported recently. As shown in Table 1, Anhauser et al.^[^ ^27^ ^]^ used aryl ketone derivatives as a new type of photocage group. By introducing aryl ketone derivatives at the N‐7 position of guanine to form purine iminium ions, the photocage of purine nucleosides was realized, and a new photocaged site that is not suitable for o‐nitrobenzene was discovered. Generally, the exocyclic O6 of guanosine is the optimal site for modifying the photocaging groups.^[^ ^49^ ^]^ While in DNA and RNA, the N7 site of guanosine is the most potent nucleophile which can rigorously affect Hoogsteen recognition site. As we know, this is the first report of photocaged nucleoside method at the N‐7 position of guanosine. In addition, Xiao et al.^[^ ^50^ ^]^ designed a new strategy for DNA photocaging through the photo‐modulation of phosphorothioate chemistry, and the excitation wavelength can be regulated by different linking groups.

**TABLE 1:** Examples of photocleavage chemistries

	Schematics	Ref.
Benzophenone	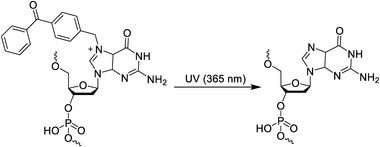	^[^ ^27^ ^]^
Phosphorothioate Chemistry	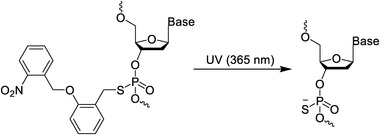	^[^ ^50^ ^]^
	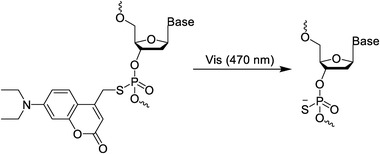	

### 2.2 Photoisomerization molecules

**FIGURE 3:**
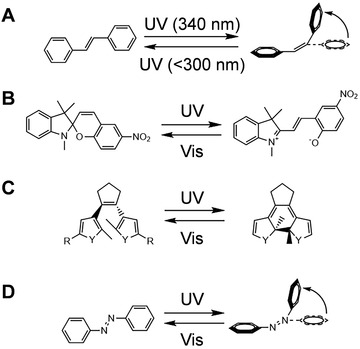
Overview of the photoisomerization of four classical photoresponsive molecules: (A) Stilbene, (B) spiropyran, (C) diarylethene, (D) azobenzene

The photoisomerization process can also reversibly control the structural changes of nucleic acid sequences. Photoisomerization molecules including stilbene, spiropyran, diarylethene, and azobenzene derivatives are the most commonly used photosensitive molecules for the reversible control of nucleic acid sequences (Figure 3).

Stilbene and its analogs are a well‐known example of the photoisomerization process. As shown in Figure 3A, under different wavelengths of ultraviolet light, stilbene can be reversibly isomerized between cis and trans forms. Lewis and Liu^[^ ^51^ ^]^ reported for the first time that this molecule was used to modify DNA nanostructures for optical control of its mechanical motion. Stilbene and azobenzene have structural similarities and show similar photochemistry characteristics.^[^ ^52^ ^]^ But the photoisomerization of stilbene requires a significant amount of space, because the rotation of the benzene ring is achieved by cis‐trans isomerization of the C‐C double bond.^[^ ^53^ ^]^ Therefore it is difficult for stilbene to perform photoisomerization reactions in double‐stranded DNA.^[^ ^54^ ^]^ In addition, the cis‐isomer of stilbene tends to undergo irreversible cyclization/oxidation, which is also the main disadvantage of stilbenes as photoisomerization molecules.^[^ ^55^ ^]^ Therefore, stilbene is rarely used in the construction of photoisomerized nucleic acid systems to control the structural changes of nucleic acid sequences. But stilbene and its derivatives can be loaded at the end of nucleic acid sequences to regulate nucleic acid functions.^[^ ^56^ ^]^

**FIGURE 4:**
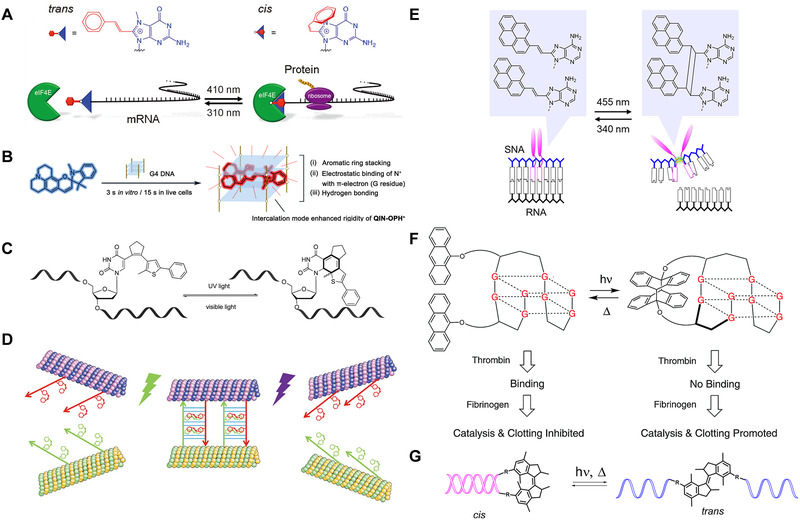
(A) Schematic diagram of light regulation of translation by cis‐trans isomerization of stilbene. Reproduced with permission.^[^ ^57^ ^]^ Copyright 2014, Wiley‐VCH Verlag. (B) Illustration of the detection of G4 DNA with spiropyran fluorescent probe. Reproduced with permission.^[^ ^59^ ^]^ Copyright 2019, The Royal Society of Chemistry. (C) Schematic of photoswitchable DNA where a pyrimidine nucleotide is part of the photoswitch. Reproduced with permission.^[^ ^64^ ^]^ Copyright 2013, John Wiley & Sons. (D) Schematic of selective association and dissociation of DNA conjugated microtubules under UV and Vis light irradiation, respectively. Reproduced with permission.^[^ ^73^ ^]^ Copyright 2018, Nature Publishing Group. (E) Scheme diagram of reversible light regulation of SNA/RNA duplex. Reproduced with permission.^[^ ^74^ ^]^ Copyright 2019, American Chemical Society. (F) Diagram of regulating the function of aptamers through the photocycloaddition reaction. Reproduced with permission.^[^ ^75^ ^]^ Copyright 2019, Nature Publishing Group. (G) The concept for motor‐DNA hybrid based on molecular motors. Reproduced under the terms of the CC‐BY‐NC‐ND license.^[^ ^76^ ^]^ Copyright 2018, American Chemical Society

Ogasawara^[^ ^57^ ^]^ developed a reversible protein expression regulation method. As shown in Figure 4A, the stilbene derivative 7‐methyl‐8‐styryl guanosine is attached to the end of the messenger RNA (mRNA). When it is in trans isomerization, the eukaryotic initiation factor 4E is difficult to bind to mRNA chain and inhibits protein synthesis. After being irradiated with 410 nm light, the molecular configuration changes to cis, allowing the promoter to bind to the mRNA chain, thereby realizing protein translation. This method provides a high on/off ratio and rapid response in terms of light‐induced protein expression and inhibition, and can regulate protein expression in living cells with precise light control methods.

Spiropyrans are organic photochromic molecules.^[^ ^58^ ^]^ As shown in Figure 3B, spiroyran can be reversibly transformed between the closed, nonplanar form and the open, planar merocyanine form under different wavelengths of light.^[^ ^59^ ^]^ In DNA nanocomponents, spiropyran exhibit enhanced photosensitivity induced by aggregation,^[^ ^60^ ^]^ and photoisomerization can occur between the colorless spiropyran state and colored merocyanine state.^[^ ^61^ ^]^

Avagliano et al.^[^ ^62^ ^]^ explored the binding mechanism of photoswitches spirogyran and DNA through computational methods. This work indicates that by modulating the electrostatic terms in spiropyran probes, such as DNA binding energy and electrostatic potential, it can provide guidelines for the design of spiropyran optical switches with specific DNA binding modes. Li and coworkers^[^ ^59^ ^]^ designed a spiropyran‐linked fluorescent probe that can detect endogenous DNA G‐quadruplexes (G4) in living cells in real time. As shown in Figure 4B, by the role of electrostatics, the fluorescent emission wavelength can be greatly red‐shifted within 3 s in vitro. After entering living cells, it can emit fluorescent within 15 s. In addition, recent studies have proved that the quinolizidine‐substituted spiropyran can specifically target G4 at physiological pH.^[^ ^63^ ^]^

As shown in Figure 3C, different wavelengths of light irradiation can reversibly change the spatial structure of diarylethene. Diarylethene has shown its promise as a scientific tool for light control systems. It was found that it undergoes an efficient and reversible electric ring rearrangement, and the switching wavelength can be adjusted by the chemical properties of the substituents.^[^ ^64^ ^]^ Diarylethene has strong anti‐fatigue ability, and its reversible light switching times can usually reach hundreds of times.^[^ ^52^ ^]^ It has been well‐studied as a photoresponsive scaffold for binding to duplex DNA.^[^ ^65^ ^]^ However, the structural change of diarylethene between open and closed forms is small, making it difficult to provide sufficient mechanical power to stimulate movement.^[^ ^23^ ^]^

Cahova and Jaschke^[^ ^64^ ^]^ reported a new type of diarylethene photoswitche with nucleosides as one of the aryl moieties. As shown in Figure 4C, the photoswitchable oligonucleotides, in which the structural components of DNA itself participate in the rearrangement of chemical bonds, and geometric shapes changes, thus forming the active part of the photoswitches, lead to new applications of reversible DNA light switches in biology, microscopy, and nanotechnology.

Azobenzene has the advantages of easy synthesis, high switching efficiency, fast switching speed, and high quantum yields, and is one of the most attractive photoisomerization molecules in nucleic acid systems.^[^ ^66^ ^]^ In addition, azobenzene and its derivatives are one of the most commonly used molecules in versatile applications, because they can make huge changes in molecular geometry and polarity through robust, fast and reversible E/Z isomerization.^[^ ^67^ ^]^ As shown in Figure 3D, trans‐azobenzene has a planar molecule and zero dipole moment. When trans‐azobenzene is irradiated with UV light, it will isomerize into the non‐planar cis isomer, with a dipole moment of 3 D.^[^ ^68^ ^]^ While the conformational change from cis to trans can be caused by blue light or heat. The photochemical properties of the azobenzene molecules can be adjusted by rational modification of the azobenzene core structure and solvent selection.^[^ ^52^ ^]^ The mechanism of the photoisomerization of azobenzene has been controversial, but recent computational studies and experimental evidence showed that it follows the hula‐twist pathway of volume‐conservation in the DNA environment.^[^ ^69^ ^]^ The molecular size and polarity similar to nucleobases make azobenzene an excellent candidate for chemical modification of nucleic acid.^[^ ^70^ ^]^ The light responsive nucleic acid modified by azobenzene can be manipulated to realize the regulation of DNA nanostructure.^[^ ^71^ ^]^ The key to light‐controlled nucleic acid nanostructures is to adjust the stability imparted by photosensitive azobenzene to achieve the hybridization/dehybridization of nucleic acid nanostructures.^[^ ^72^ ^]^

Keya and coworkers^[^ ^73^ ^]^ designed a DNA functionalized microtubule in which azobenzene molecules are embedded in the DNA phosphate backbone. As shown in Figure 4D, DNA containing azobenzene groups can respond to stimulation of visible (Vis) light or UV light, so that the DNA‐modified microtubules can switch between solitary and swarm behavior. The capabilities of such supramolecular systems can be programmed and controlled by the swarming behavior of the DNA‐functionalized microtubules. When the azobenzene is in cis isomerism, the non‐planar azobenzene molecules make it difficult to hybridize between DNA sequences, causing microtubules to move in solitary. Under visible light irradiation, azobenzene isomerizes from cis form to trans form, which hybridizes DNA strands with the photo‐isomeric molecules inserted and makes microtubules swarm. After UV exposure, the microtubules return to their original state. This feature can successfully achieve the repeated and reversible switching of the swarm behavior of microtubes with the light of different wavelengths.

In addition to the above‐mentioned four classic photoisomerization molecules, researchers also designed some other photoisomerization molecules. For example, Murayama and coworkers^[^ ^74^ ^]^ designed a photoresponsive 8‐pyrenylvinyl adenine molecule as a substituted nucleotide to play a role in serinol nucleic acid (SNA). As shown in Figure 4E, under 450 nm wavelength light, the adjacent 8‐pyridines will undergo intermolecular [2+2] photocyclization reaction, which will twist the SNA chain and cause the dissociation of the duplex SNA/RNA, and the duplex is almost completely dissociated. Upon 340 nm light irradiation, the SNA/RNA restored duplex formation by cycloreversion. These reversible adjustment methods have the characteristics of high yield, rapid reaction, and high selectively. To our knowledge, it is the first example of reversible light regulation of nucleic acid duplex hybridization and dehybridization through photocycloaddition and cycloreversion at room temperature.

In addition, Ali and coworkers^[^ ^75^ ^]^ designed a photoresponsive thrombin binding aptamer by modification with two anthracene groups. When the light‐responsive aptamer is not triggered, the aptamer binds to thrombin and inhibits the coagulation process. Under UV light irradiation, anthracene undergoes photodimerization, which distorts the aptamer structure, inhibits the combination of the aptamer and thrombin, and triggers the catalysis effect and the resulting coagulation process (Figure 4F). After heat treatment, the structure of the aptamer and the ability to bind to thrombin can be restored. This strategy provides ideas for intelligent anticoagulation therapy.

Lubbe's group^[^ ^76^ ^]^ designed a photoresponsive molecule as a molecular motor through computer‐aided molecular design technology, and embedded it as a backbone molecule into the DNA nucleic acid backbone to control the hybridization and dehybridization of an 8‐mer DNA strands. As shown in Figure 4G, the hairpin formation is not affected when the photoresponsive molecules are in stable trans isomerism. Under UV light irradiation, the photoresponsive molecules rotate, and this rotation will produce larger structural changes that dissociate the DNA double‐stranded structure. After further exposure to light and heat, the light responsive molecule can restore its trans isomerism, allowing DNA to rehybridize and restore the hairpin structure.

### 2.3 Photocrosslinking molecules

**FIGURE 5:**
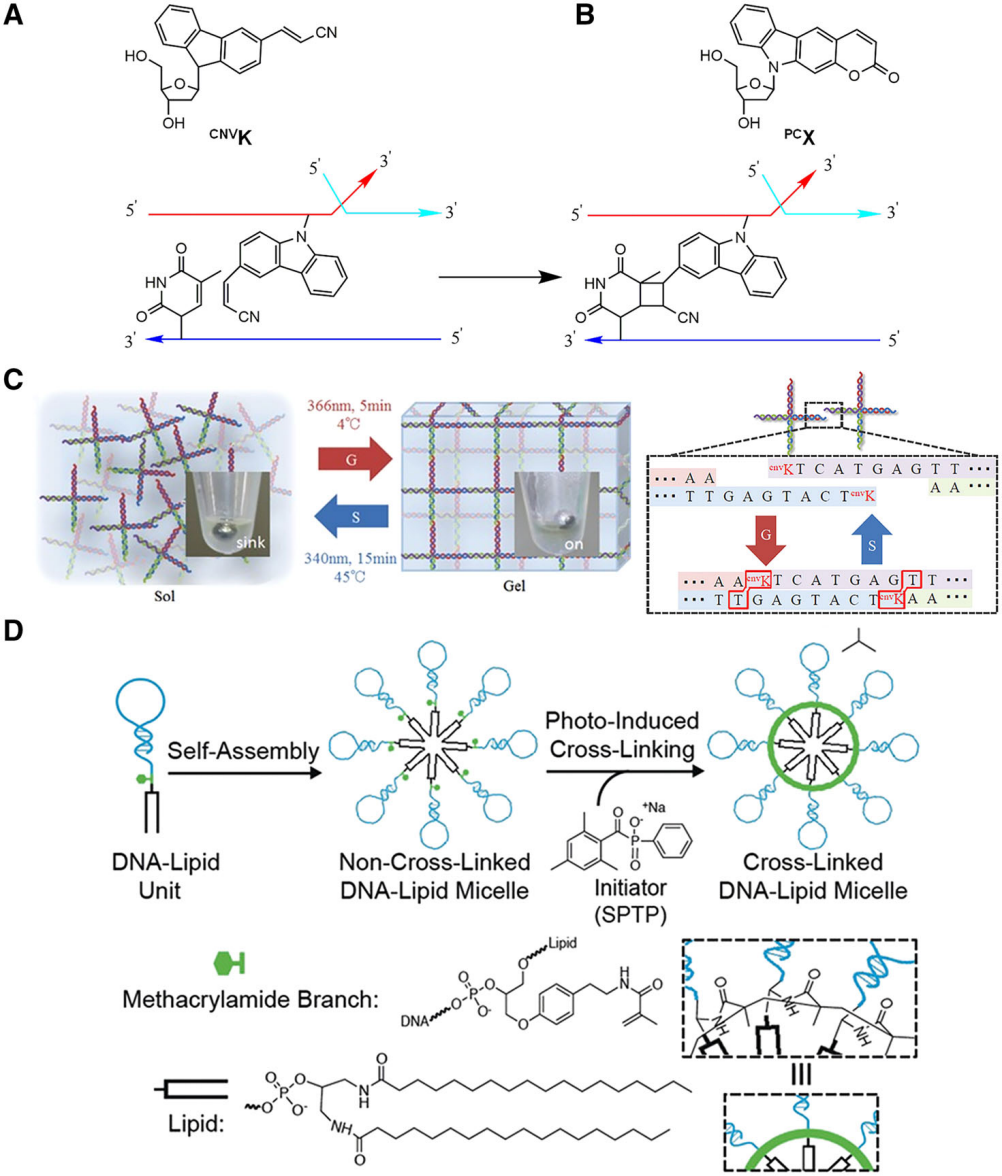
(A) Structure of ^CNV^K (A) and ^PC^X (B) and the working principle of photocrosslinking reaction. Adapted with permission.^[^ ^79^ ^]^ Copyright 2018, American Chemical Society. (C) The working principle of gel‐sol transition of X‐motif modified with ^CVN^K. Reproduced with permission.^[^ ^83^ ^]^ Copyright 2016, Wiley‐VCH Verlag. (D) Schematic diagram of self‐assembly and light‐induced crosslinking of DNA‐lipid monomers. Reproduced with permission.^[^ ^84^ ^]^ Copyright 2018, John Wiley & Sons

Reversible light responsive nucleic acid crosslinking can perform manipulation on the sequence of nucleic acid to realize the regulation of nuclear nucleic acid structure and sequence‐specific detection.^[^ ^77^ ^]^ Photocrosslinking molecules are often used in the construction or stabilization of DNA nanostructures. The mechanism of photocrosslinking between nucleic acid strands is shown in Figure 5A. The photocrosslinking groups and the corresponding groups of the nucleic acid bases undergo a photocrosslinking reaction, so that the ends of two adjacent strands can be connected together. 3‐cyanovinylcarbazole nucleoside (^CNV^K) (Figure 5A) is one of the most commonly used photocrosslinking groups. Under the irradiation of 366 nm UV light irradiation, it can undergo a [2+2] cycloaddition reaction with the pyrimidine bases to realize ultrafast photocrosslinking between complementary strands. This ultrafast reversible photocrosslinking can be effectively used in biotechnology and nanotechnology, especially in the photochemical regulation of site‐specific nucleic acid structures.^[^ ^78^ ^]^ However, this photocrosslinking reaction requires exposure to UV light for several seconds, which will lead to increased phototoxicity.^[^ ^79^ ^]^

Fujimoto and coworkers^[^ ^79^ ^]^ developed a novel photocrosslinker pyranocarbazole nucleoside with a wide π‐conjugate structure (Figure 5B). Under 400 nm light, the crosslinking site on the furan ring of the photocrosslinking molecule can be crosslinked with the pyrimidine base through the [2+2] cycloaddition reaction without any side reaction. Compared with the UV‐A light irradiation used in ^CNV^K, the excitation light wavelength of this photocrosslinking agent is longer and the excitation time is shorter. These characteristics make it have lower phototoxicity for practical applications, which makes it effective in adjusting, and has great potential in the detection and manipulation of nucleic acids in living cells.

In recent years, photo‐crosslinking molecules have been introduced into hydrogels by many researchers. Hydrogels have attracted increasing interest in the fields of biosensing, biomedical tissue engineering, and functional materials.^[^ ^80^ ^]^ Some hydrogel matrices have stimulus responsiveness, which can undergo phase conversion between gel and liquid under external stimuli.^[^ ^81^ ^]^ This phase change can occur in the presence of disease biomarkers or a specific physiological environment.^[^ ^82^ ^]^ Therefore, these functional hydrogels have great potential for functional sensing, drug delivery, and tissue repair. Nucleic acid nanostructures containing photocrosslinking molecules are good candidates for the construction of phase transitions hydrogels. Kandatsu and colleagues^[^ ^83^ ^]^ designed an X‐shaped DNA motif. At the tip of each arm of the X‐shaped motif, photocrosslinking groups 3‐cyanovinylcarbazole (^CNV^K) are introduced. As shown in Figure 5C, this gel provides repeatable sol‐to‐gel and gel‐to‐sol transitions, which are driven by ultraviolet radiation with different wavelengths and temperatures. Under 366 nm UV light irradiation, the sticky ends of the X‐shaped motifs hybridize to each other and form a gel along with the cross‐linking of ^CNV^K molecules. As a result, the gel system undergoes a transition from sol to gel. Under the light of 340 nm, the connection between ^CNV^K molecules was broken, and the connection between the X motifs was in a metastable state. Leading to its dehybridization to a monomer at 45°C. The gel system undergoes a transition from gel to sol.

In addition, photo‐crosslinking molecules can also be used to stabilize DNA micelles. Li and coworkers^[^ ^84^ ^]^ designed crosslinked aptamer‐lipid micelles. Between the hydrophilic and hydrophobic domain of this lipid‐based DNA micelle monomer, a methacrylamide functional group is introduced. As shown in Figure 5D, after the micellar monomers are self‐assembled in aqueous solution, the introduced methacrylamide groups can undergo radical polymerization under UV light to connect together. Compared with non‐crosslinked micelles, the photocrosslinked DNA micelles bave improved stability in the cellular environment. And when the micelle binds to the target, it can further provide excellent cell recognition specificity.

### 2.4 Two‐photon excitation molecules

Most photosensitive molecules usually respond to UV or Vis light and then undergo structural changes. However, UV or Vis light has poor permeability to deep tissues. So photosensitive molecules can only be applied to surface tissues^[^ ^85^ ^]^ or controlled by invasive fiber optic implants, which has great limitations in clinical application.^[^ ^86^ ^]^ To solve this problem, near‐infrared (NIR) light has become one of the most attractive candidates because it can penetrate deep tissues and cause minimal phototoxicity.^[^ ^87^ ^]^

**FIGURE 6:**
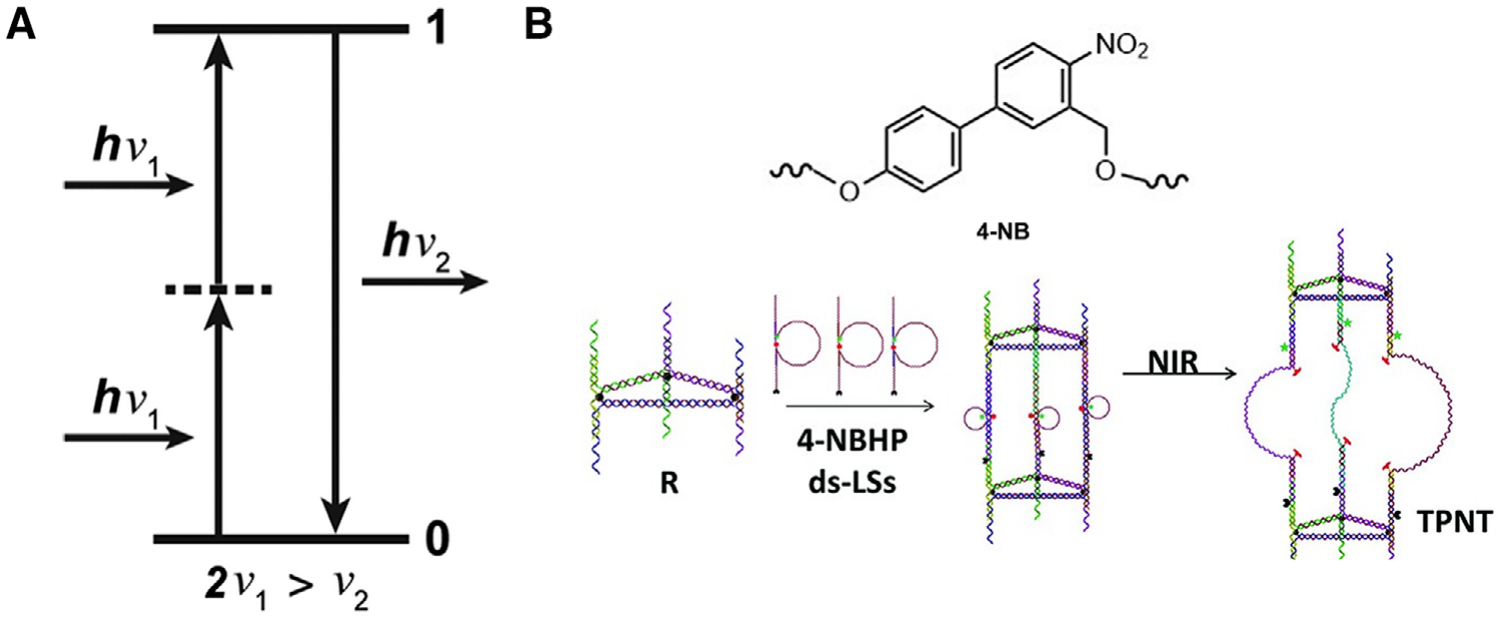
(A) Schematic diagram of two‐photon absorption. Reproduced with permission.^[^ ^33^ ^]^ Copyright 2017, Royal Society of Chemistry. (B) The 4‐NB structural formula and linear TPNT self‐assembly and its two‐photon photocleavage under near‐infrared excitation. Reproduced with permission.^[^ ^89^ ^]^ Copyright 2015, Wiley‐VCH Verlag

The two‐photon excitation process involves photosensitive molecules that can absorb two photons at the same time to reach a higher energy level. Then the electron transitions back to the ground state and release photons. The energy of released photons is higher than that of absorbed photons (Figure 6A). An unstable virtual state will appear during this process. But this kind of intermediate state does not exist, resulting in low two‐photon excitation efficiency, which requires a femtosecond pulsed lasers to achieve.^[^ ^33^, ^88^ ^]^ Generally, photosensitive molecules with maximum absorption above 350 nm can be excited by NIR or IR light (700∼1000 nm).^[^ ^23^ ^]^

Dai and coworkers^[^ ^89^ ^]^ designed and synthesized a 4‐nitro‐4′‐phenoxy‐1,1′‐biphenyl (4‐NB) molecule (Figure 6B) and integrated it into triangular DNA nanotubes through covalent bonds. As shown in Figure 6B, two‐photon‐responsive DNA nanotubes (TPNT) are assembled through a layered modular approach. Under the irradiation of NIR femtosecond laser, the 4‐NB molecules undergo photocleavage along the three sides of the TPNT, resulting in the opening of the tube cavity and part of the single‐stranded nanotubes. This will cause the nanotube to transform from a linear conformation to a curved conformation. FBS incubation experiments confirmed that the linear conformation can effectively protect TPNT from nuclease degradation. Hela cell uptake experiments showed that TPNT in the linear conformation has great cell uptake and internalization, while TPNT in the curved conformation can only attach to the cell surface. These characteristics are conducive to their use as suitable smart nanocarriers for drug delivery.

## 3 POSITION OF THE MOLECULES IN THE NUCLEIC ACID

**FIGURE 7:**
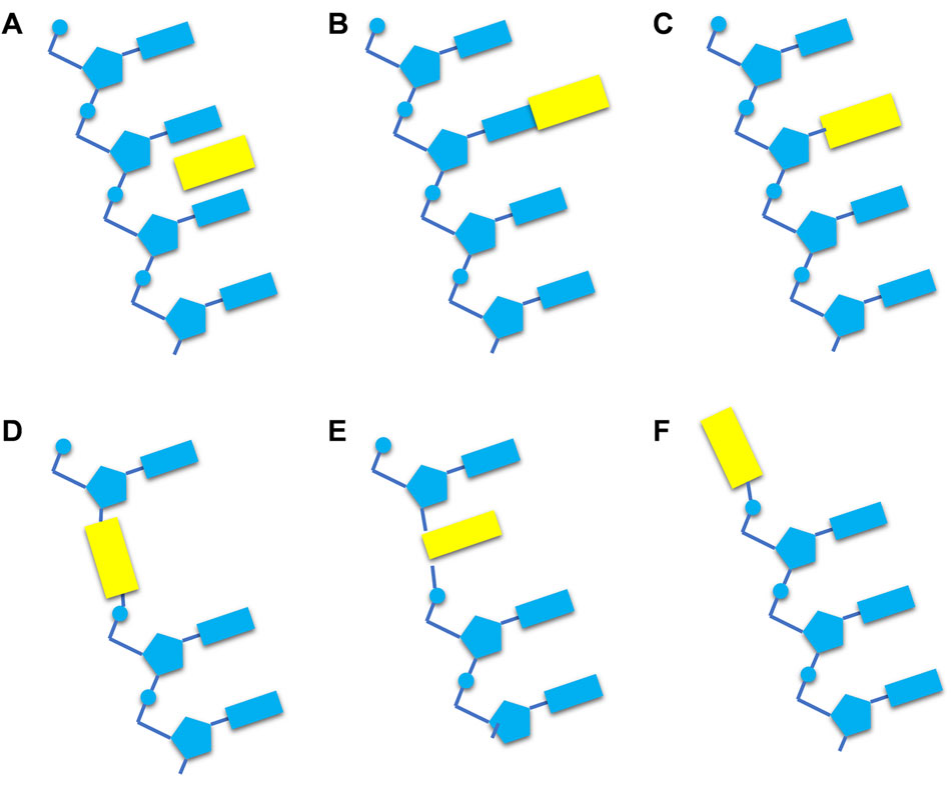
Schematic diagram of the six positions of light‐responsive molecules in nucleic acids: (A) noncovalent interactions, (B) attachment to nucleobases, (C) substituted nucleobases, (D) backbone insertion, (E) substituted nucleotides, (F) end capping. Yellow rectangles represent photoresponsive molecules. Adapted with permission.^[^ ^52^ ^]^ Copyright 2017, Royal Society of Chemistry

The photoresponsive molecules have different effects at different positions in the nucleic acid strands. As shown in Figure 7, this article introduced six positions of photoresponsive molecules in nucleic acids that are used to regulate nucleic acids. Including noncovalent interactions (Figure 7A), attachment to nucleobases (Figure 7B), substituted nucleobases (Figure 7C), backbone insertion (Figure 7D), substituted nucleotides (Figure 7E), and end capping (Figure 7F).^[^ ^52^ ^]^

### 3.1 Noncovalent interactions

Figure 7A shows the position where the light responsive molecules regulate nucleic acid structures through non‐covalent interactions. This method is usually realized by binding light‐responsive molecules with cationic tails negative charges in nucleic acid sequences, and can be used to regulate the hybridization of nucleic acid sequences.^[^ ^90^ ^]^

**FIGURE 8:**
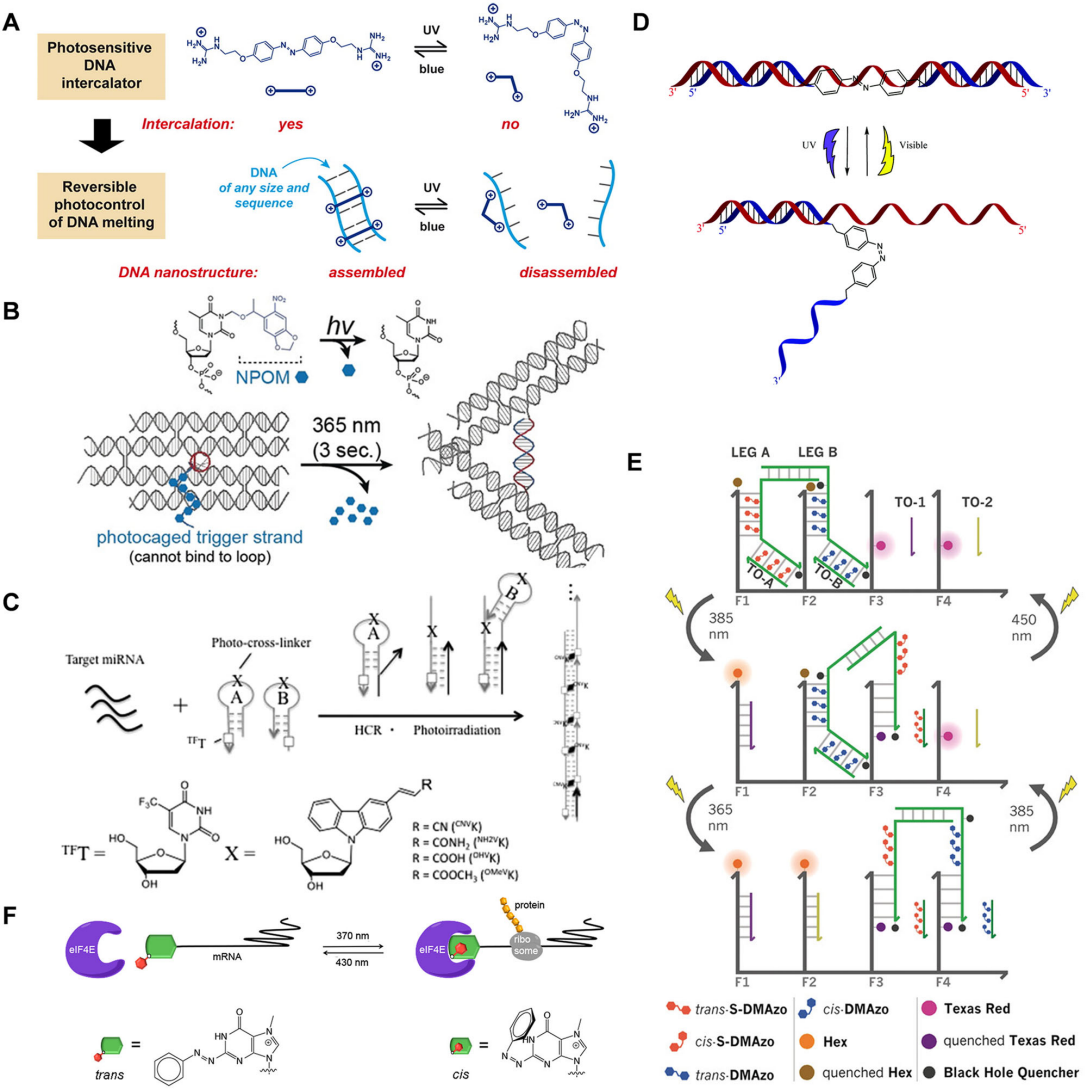
(A) The cis‐trans isomerization of AzoDiGua molecule and its regulation of oligonucleotide double‐strand hybridization under different wavelengths of light. Reproduced with permission.^[^ ^91^ ^]^ Copyright 2016, American Chemical Society. (B) Schematic diagram of DNA tweezers controlled by light cage/uncage. Reproduced with permission.^[^ ^94^ ^]^ Copyright 2018, John Wiley & Sons. (C) Schematic diagram of 19F NMR‐based miRNA detection using hybrid chain reaction. Reproduced with permission.^[^ ^96^ ^]^ Copyright 2018, Royal Society of Chemistry. (D) Schematic diagram of reversible light control of siRNAzos. Reproduced with permission.^[^ ^100^ ^]^ Copyright 2019, Royal Society of Chemistry. (E) The working principle of a DNA walker modified by two different azobenzene derivatives. Reproduced with permission.^[^ ^101^ ^]^ Copyright 2019, John Wiley & Sons. (F) Schematic diagram of the reversible light regulation of protein translation by the light‐responsive cap. Reproduced with permission.^[^ ^102^ ^]^ Copyright 2017, American Chemical Society

Bergen and coworkers^[^ ^91^ ^]^ synthesized a photoresponsive molecule (AzoDiGua) for non‐covalent insertion into DNA double strands. As shown in Figures 8A, the photoresponsive molecule consists of a photoisomerizable azobenzene molecule and two terminal guanidine functional cationic tails. When the azobenzene moiety is in trans isomerism, AzoDiGua enters the DNA double‐strand through electrostatic interaction, and can stabilize the DNA double‐strand structure through π stacking, so that the melting temperature is significantly increased. Under UV light irradiation, the trans‐AzoDiGua molecule isomerize to non‐planar cis‐isomer, which is not conducive to the π stacking and reduces the melting temperature of DNA double‐strand. Under the excitation of blue light, the melting temperature can be restored to the original level. This highly dynamic and reversible melting temperature adjustment method can regulate the hybridization and dehybridization of complementary oligonucleotides at constant temperature. In addition, Lafon et al.^[^ ^92^ ^]^ designed a method to produce light‐switchable coacervate droplets through the phase separation process of short DNA duplex through azobenzene cations. Such droplet can switch between dissolution and aggregation under different wavelengths of light, and has potential in simulating the dynamic structure of membraneless organelles.

### 3.2 Attachment to nucleobases

Figure 7B shows a method in which photoresponsive molecules are connected to nucleic acid bases via bridging groups such as ether bonds and ester bonds to regulate the structure of nucleic acids.^[^ ^52^ ^]^ Photoresponsive molecules are often used as irreversible photocage molecules. When caged molecules are attached to nucleobases, it is difficult for the n nucleobases to pair with each other through Watson‐Crick hydrogen bonds. After being illuminated, the photocage molecules break away from the nucleobases, restore the complementary pairing ability of the nucleic acid bases, and then restore the function of the nucleic acids.^[^ ^93^ ^]^

Liu et al.^[^ ^94^ ^]^ designed a fast and irreversible DNA nanostructure switching strategy. As shown in Figure 8B, the trigger sequence used to control the switching of DNA nanostructure is incorporated into the DNA structure from the beginning. However, photocage molecules are attached to the nucleobases of the trigger strand before these sequences are excited, making it difficult to hybridize with the complementary strand. When the DNA nanostructure is illuminated by 365 nm light, the photocage molecules break away from the nucleobases of the trigger strand, restore their hybridization function, and trigger the switching of the DNA nanostructure. Compared with external stimulus triggering, this DNA nanostructure switching method has the advantages of faster response speed and higher time and space accuracy.

### 3.3 Substituted nucleobases

Figure 7C shows a method for light‐responsive molecules to control nucleic acid structure by substituting nucleobases. Photoresponsive molecules substituted for nucleobases have no significant effect on the hybridization ability of nucleic acid sequences, such as photocrosslinking molecules.^[^ ^95^ ^]^ Under light irradiation, they can undergo a photocrosslinking reaction with pyrimidine bases to connect the nucleic acid motifs together. This method can control the aggregation of nucleic acid motifs, such as controlling the switching of DNA motifs between gel and sol.^[^ ^83^ ^]^

Besides, Nakamura and coworkers^[^ ^96^ ^]^ developed a nucleic acid sequence‐specific detection method. As shown in Figure 8C, the specific nucleic acid sequence detection system consists of two DNA hairpin probes (probe A and probe B). The photocrosslinking group 3‐vinylcarbazole derivatives are introduced into the loops of DNA hairpin probes A and B, respectively. The end of probe A is modified with trifluorothymidine, which can be detected by ^19^F NMR. When the targeted miRNA is present, it induces the strand displacement reaction of probe A and opens the hairpin, exposing a new sticky end complementary to probe B to open the hairpin of probe B, which has the same sticky end as the target miRNA sequence. In this way, each copy of the target miRNA can induce a alternating hybridized chain reaction between the probe A and probe B to amplify the miRNA signal. Under light irradiation, trifluorothymidine and the photocrosslinking group undergo a cycloaddition reaction, which induces a large chemical shift. This method can detect target miRNAs as low as 10 nM in a sequence‐specific manner.

### 3.4 Backbone insertion

The structure of nucleic acid can be adjusted by introducing photoresponsive molecules through backbone insertion (Figure 7D), such as photocleavage molecules and photoisomerization molecules. Some molecules can regulate reactions, such as photocleavage molecules and photoisomerization molecules. The former can induce long sequences to be light‐cut into short sequences, leading to strand displacement reactions.^[^ ^44^ ^]^ The latter can affect the hybridization and function of nucleic acid strands through reversible photoisomerization.^[^ ^97^ ^]^ This combinatorial strategy can be used to regulate multiple biological activities, such as gene editing,^[^ ^44^ ^]^ bioimaging,^[^ ^98^ ^]^ and nuclease activation,^[^ ^99^ ^]^ etc.

Hammill and coworkers^[^ ^100^ ^]^ designed a small interfering RNA (siRNA) with azobenzene embedded in the backbone (siRNAzo) to reversibly control RNA interference. As shown in Figure 8D, under Vis light irradiation, the azobenzene molecule is in trans isomer, so that the sense strand containing azobenzene is hybridized with the antisense strand, thereby activating the RNA interference activity of siRNAzo. Under UV light irradiation, azobenzene switches to non‐planar cis‐isomer, which reverses the sense strand structure and causes inactivation of siRNAzo. This strategy can perform real‐time light control of different silencing siRNAs, which is beneficial for controlling adverse side effects in susceptible individuals and reducing the unexpected side effects caused by siRNA.

### 3.5 Substituted nucleotides

The method of substituted nucleotides is shown in Figure 7E. It is one of the most effective methods for regulating the hybridization and dehybridization of nucleic acid sequences. In this method, photoresponsive molecules such as azobenzene derivatives are incorporated into the nucleic acid chain via a diol linker. Unlike backbone insertion, photoresponsive molecules substituted for nucleotides can play a base‐like role through π‐π conjugation.^[^ ^52^ ^]^

Skugor and coworkers^[^ ^101^ ^]^ designed a light‐activated DNA walker that can move bidirectionally on a one‐dimensional path. As shown in Figure 8E, two azobenzene derivatives (S‐DM‐Azo and DM‐Azo) are inserted into single‐stranded DNA via d‐threonine as the two legs of the DNA walker. And the two molecules can control the DNA strand displacement reaction through cis‐trans isomerization. The four footholds on the path of the walker are all modified with fluorophores. Under different wavelengths of light, the forward and backward movement of the DNA walker can be controlled, and the position of the walker can be indicated by the quenching of two different fluorescent groups. The movement and position of each step of this light‐activated non‐autonomous non‐random DNA walker can be controlled with high precision, and it is hopeful that it will become an important tool in DNA nanotechnology.

### 3.6 End‐capping

Figure 7F shows a nucleic acid modified with photoresponsive molecules end‐capping. Since the position of the light‐sensitive molecule is at the end of the nucleic acid strand rather than the middle, it is difficult for the photoresponsive molecule to change the structure of the nucleic acid strand after being exposed to light. After light irradiation, the photoresponsive molecule can be completely separated from the nucleic acid or photoisomerized, leading to different applications.

Ogasawara^[^ ^102^ ^]^ introduced an azobenzene derivative into the 5′‐end of mRNA and developed a reversible protein expression regulation method. As shown in Figure 8F, when the photoresponsive molecule is in trans isomerism, the exposed benzene ring produces a large steric hindrance, which makes it difficult for the initiation factor eIF4E to bind to the mRNA strand and inhibit protein synthesis. After being irradiated with 370 nm light, the molecular configuration changes to cis, allowing the promoter to bind to the mRNA strand, thereby realizing protein translation. When exposed to 430 nm light, the photoresponsive molecules can be converted from cis to trans isomers, thereby shutting down protein translation. Compared with the trans isomer, the translated protein yield of the cis isomer is 7.1 times higher than that of the trans isomer. This method can light‐induce the development of double‐headed zebrafish by controlling the expression cycle of strabismus. The structural details of the different binding sites in the above examples are shown in Table 2.

**TABLE 2:** Examples of linkage methods between light‐responsive molecules and nucleic acids

Linkage methods	Structural illustration	Photo‐induced structural changes	Ref.
Noncovalent interactions	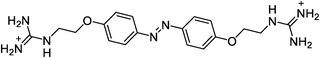	Hybridization/dehybridization	^[^ ^91^ ^]^
Attachment to nucleobases	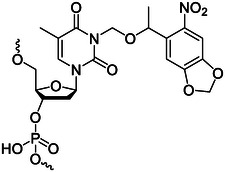	Hybridization	^[^ ^94^ ^]^
Substituted nucleobases	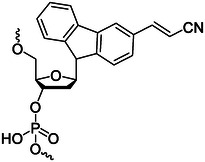	Photocrosslinking	^[^ ^96^ ^]^
Backbone insertion	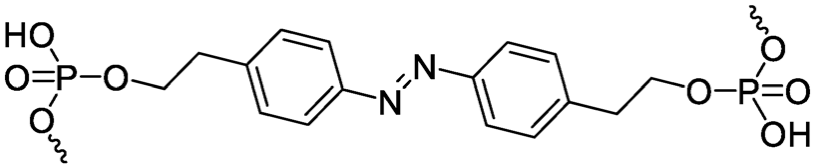	Strand flip	^[^ ^100^ ^]^
Substituted nucleotides	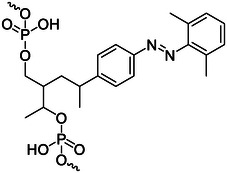	Hybridization/dehybridization	^[^ ^101^ ^]^
End‐capping	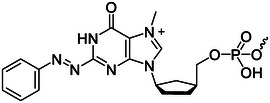	elF4E affinity/non‐affinity	^[^ ^102^ ^]^

## 4 BIOLOGICAL APPLICATIONS

The temporal and spatial controllability of light responsive nucleic acid and the diversity of modification sites make it an excellent design module to construct various nanomachines and realize multiple applications. In addition, the low immunogenicity and good biocompatibility of nucleic acid make it one of the strong candidates for biomedicine. For example, the photo‐induced structural changes of light responsive nucleic acids make them good drug carriers and biosensors. As a natural genetic material, light responsive nucleic acid is a homolog of the genetic material in the cell and is easier to interact with it. This has great advantages in gene editing and regulating the activities of living organisms. Therefore, how to rationally design nucleic acid for its biomedical applications has been a concern of researchers. The following will briefly introduce the applications of drug delivery, biosensing, optogenetics, and gene editing.

### 4.1 Drug delivery

Nucleic acid is the genetic material in nature, which is biocompatible and biodegradable. Therefore, the nucleic acid vector can provide the maximum effect with the least toxicity, and it is a highly potential drug delivery carrier.^[^ ^103^ ^]^ Light responsive nucleic acid drug delivery systems which can remotely control the release of drugs in a time‐ and space‐controllable manner, and received extensive attention and research.

**FIGURE 9:**
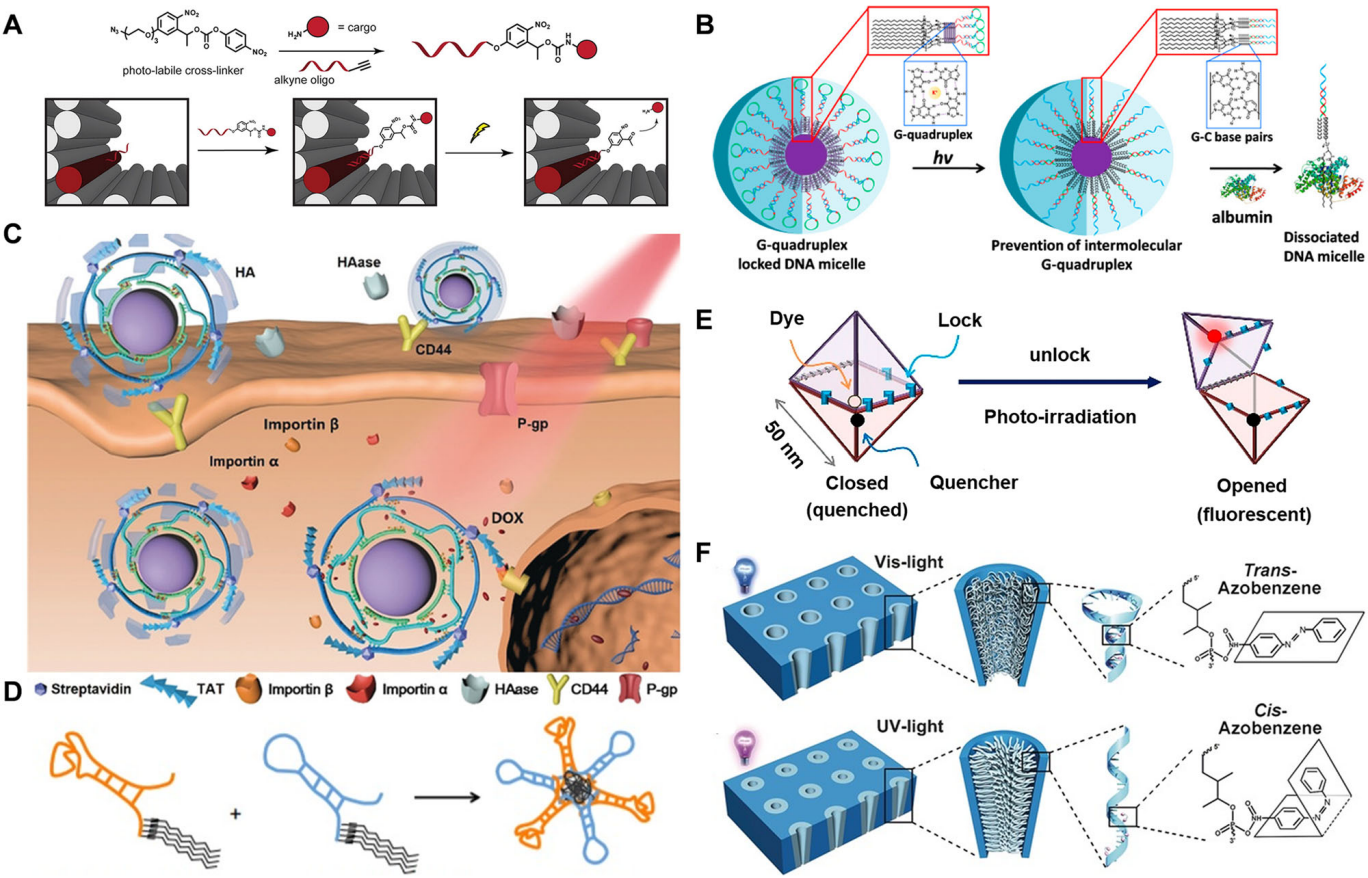
(A) Schematic diagram of cargo encapsulation, photo‐cracking reaction, and cargo release. Reproduced with permission.^[^ ^104^ ^]^ Copyright 2016, American Chemical Society. (B) Light‐controlled process of DNA micelles with adjustable stability. Reproduced with permission.^[^ ^105^ ^]^ Copyright 2017, American Chemical Society. (C) Working principles diagram of light‐controlled DNA nanopump. Reproduced with permission.^[^ ^106^ ^]^ Copyright 2019, John Wiley & Sons. (D) Schematic diagram of trCLN3‐L4 and DxR‐L4 and the self‐assembly of the two domains. Reproduced with permission.^[^ ^107^ ^]^ Copyright 2018, Nature Publishing Group. (E) Schematic diagram of light‐controlled cargo release from DNA nanocapsules. Reproduced with permission.^[^ ^108^ ^]^ Copyright 2020, John Wiley & Sons. (F) Schematic diagram of light‐controlled Azo‐DNA modified nanochannel. Reproduced with permission.^[^ ^110^ ^]^ Copyright 2016, John Wiley & Sons

Kohman and coworkers^[^ ^104^ ^]^ reported a drug delivery strategy that used photoresponsive molecules as end‐capping. As shown in Figure 9A, the light responsive linkers attache the amino‐containing cargo molecules to the cavity of the DNA nanostructure. Under 500 nm light irradiation, the connection between the drug and the photocleavage group is broken, allowing the drug to be released from the end of the nucleic acid. This method can release the cargo through photocleavage in the original state, and there is no residual chemical residue. Most peptides, proteins, and bioactive molecules containing amino residues are suitable for this method, providing a universal method for the accurate release of a variety of bioactive molecules.

Jin and coworkers^[^ ^105^ ^]^ designed DNA micelle with adjustable stability using photo‐controlled dissociation of intermolecular G‐quadruplexes (Figure 9B). The G‐quadruplex structure gives DNA micelles with strong structural stability and can resist the destruction of serum albumin. A photocleavage molecule is inserted into the DNA hairpin loop as the nucleic acid backbone. Once exposed to light, the photoresponsive molecule breaks, opening the hairpin loop, and releasing the C‐rich sequence. As a result, the formation of G‐quadruplexes is blocked by strand hybridization. This causes the DNA micelles to lose stability in the event of cellular uptake and serum albumin interference. Such biocompatible DNA micelle with controllable stability has potential for in vivo drug delivery.

Zhang and coworkers^[^ ^106^ ^]^ reported a light‐driven DNA nanopump modified with azobenzene, which can release drugs quickly and efficiently. As shown in Figure 9C, DNA nanopumps are assembled by upconversion nanoparticles (UCNPs) and DNA strands modified with azobenzene. Among them, azobenzene molecules are embedded in the DNA in form of substituted nucleotides, and by embedding DOX into the DNA double helix, effective loading of DOX can be achieved. Under NIR light irradiation, UCNPs can convert NIR light into UV light and Vis light. Two different wavelengths of light can excite the azobenzene molecule to continuously switch between cis and trans isomerization, trigger alternate hybridization and dehybridization of DNA, and induce the release of DOX.

Prusty and coworkers^[^ ^107^ ^]^ designed a multifunctional light‐switchable hybrid aptamer nanostructure. As an effective molecular carrier, DNA nanostructures can selectively deliver high‐dose of DOX to cMet‐expressing cells and release payloads under light. As shown in Figure 9D, the nanocarrier is composed of a targeting domain (trCLN3‐L4) and a drug‐loading domain (DxR‐L4). The binding domain consists of four C12‐modified cMet binding aptamers. The drug‐loading domain is composed of a GC‐rich lapidated DNA hairpins to load DOX and multiple azobenzene derivatives are introduced. The recognition domain allows cMet‐expressing cells to take up and internalize the nanocarrier. Under UV light irradiation, the azobenzene molecule in the drug‐loading domains undergo isomerization, resulting in the drug release.

Tohgasaki et al.^[^ ^108^ ^]^ designed a DNA nanocapsule as a delivery vehicle for targeting cells, which could control the mechanical movement of the system through light. As shown in Figure 9E, the photocleavable DNA strand (PC) is bound to square bipyramid DNA nanocapsule (NC). When there is no light, the NC is in a locked and closed form. Upon light irradiation, the PC chain is broken, thereby opening the NC and releasing the internal cargo. This cage‐like NC system can be used as a controllable delivery carrier similar to the natural virus system to deliver large nanomaterials and proteins.

In addition to nucleic acid nanostructures as drug delivery vehicles, light‐responsive nucleic acid systems can also deliver drugs through gated formation. Li and colleagues^[^ ^109^ ^]^ assembled a system based on light‐responsive DNA aptamers to selectively and repeatedly capture and release ATP. The key functional components of the system are achieved by introducing the photoresponsive azophenyl group into ATP aptamers. In addition, Li and colleagues^[^ ^110^ ^]^ designed a light‐controlled nanochannel to modulate ion transport. As shown in Figure 9F, azobenzene molecules are embedded in single‐stranded DNA to construct a switchable control unit (Azo‐DNA) as a light‐controlled gate. By irradiating with visible light (450 nm), azobenzene molecules can be transformed into trans isomers, inducing DNA strands to transform into hairpin structures and opening the DNA channel. Under ultraviolet (365 nm) irradiation, the azobenzene molecule transforms into a cis structure, which restores the DNA chain to a relaxed state and closes the DNA channel. By alternate irradiation of Vis light and UV light, the DNA strand can be switched between folded and relaxed states to realize the opening and closing of the Azo‐DNA channel for photo‐controlled drug release and various biotechnological applications.

### 4.2 Bioimaging

The specific and sensitive detection of target RNAs plays a great role in the fields of biomedicine, new drug research, and disease diagnosis and treatment.^[^ ^111^ ^]^ Based on the unique pair recognition ability of nucleic acids, researchers have developed many RNA imaging strategies. Selective activation of RNA imaging with light can improve the spatiotemporal resolution of the imaging system.^[^ ^112^ ^]^

**FIGURE 10:**
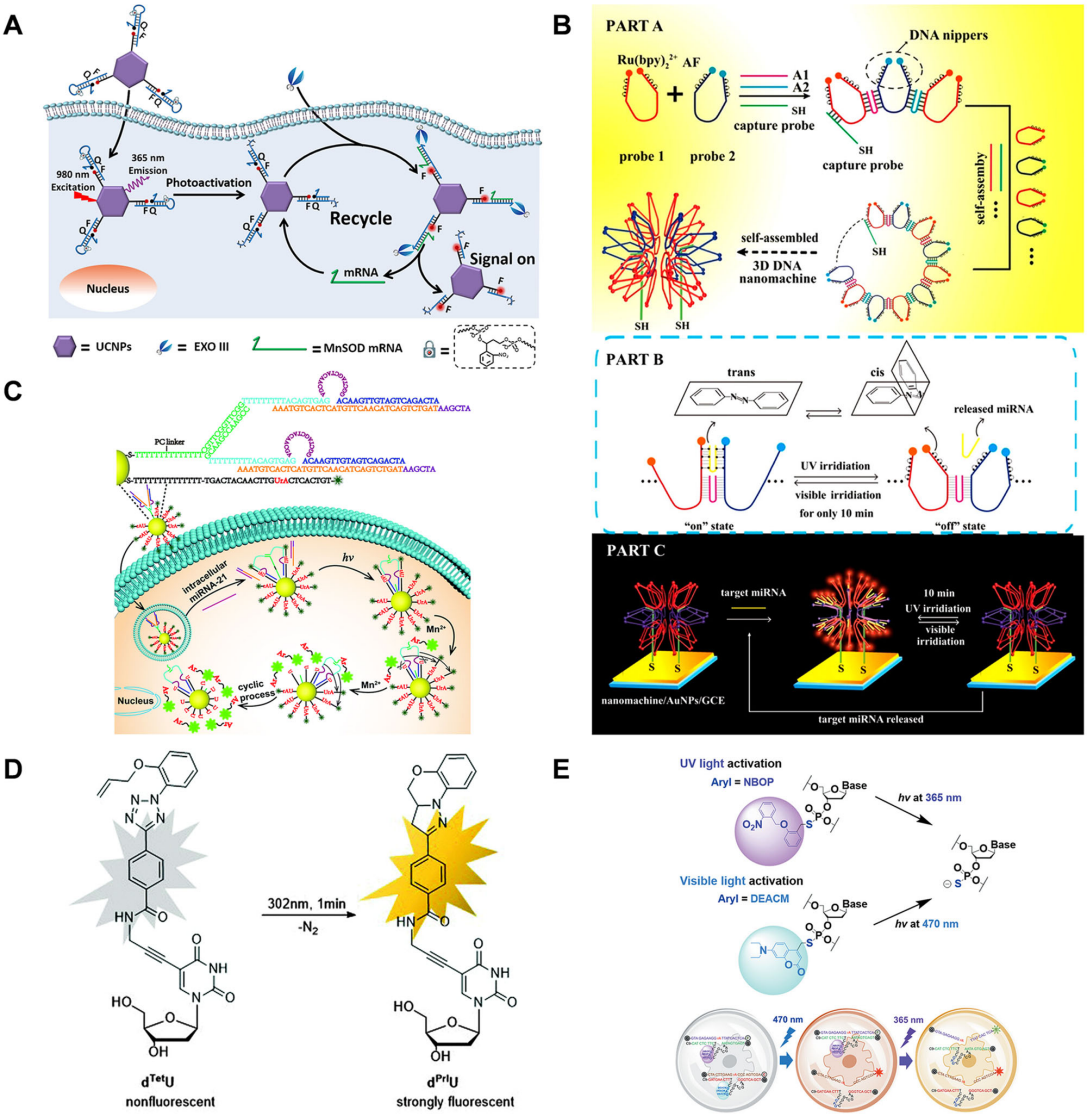
(A) Schematic diagram of near‐infrared light‐controlled cascade amplification imaging. Reproduced with permission.^[^ ^113^ ^]^ Copyright 2020, American Chemical Society. (B) Schematic diagram of the self‐assembly of 3D nanomachines (part A), the light regulation of azobenzene molecules (part B), and the rapid identification of miRNA (part C). Reproduced with permission.^[^ ^114^ ^]^ Copyright 2018, American Chemical Society. (C) Schematic diagram of miRNA‐21 starting DNA walker operation in living cells. Reproduced with permission.^[^ ^115^ ^]^ Copyright 2020, Nature Publishing Group. (D) Schematic diagram of light‐induced intramolecular cycloaddition of d^Tet^U. Reproduced with permission.^[^ ^116^ ^]^ Copyright 2016, Nature Publishing Group. (E) Schematic diagram of intracellular imaging of two DNAzymes activated different wavelengths. Reproduced with permission.^[^ ^50^ ^]^ Copyright 2021, American Chemical Society

Duan and coworkers^[^ ^113^ ^]^ designed a light‐driven mRNA imaging system and performed the nucleic acid cascade amplification reaction with the help of exonuclease III (EXO III) to amplify the mRNA signal. As shown in Figure 10A, the inactive system will not interact with the target mRNA unless it is activated by NIR light at a preset time and position. When the imaging system reaches the target site and is excited by NIR light, the UPNCs absorb the NIR light and emit UV light, cutting off photocleavage molecules embedded in DNA hairpin loops. Subsequently, the exposed recognition domains can undergo a strand displacement reaction with mRNA, and perform cascade amplification with the assistance of EXO III, to perform mRNA imaging with high sensitivity and high temporal and spatial resolution.

Zhang and coworkers^[^ ^114^ ^]^ designed a DNA nanomachine for miRNA imaging (Figure 10B). The DNA nanomachine consists of 4 single‐stranded DNAs, including probes 1, 2, and connecting strands A1 and A2. Both ends of probe 1 are labeled with an enzyme‐free electrochemiluminescence (ECL) emitter Ru(bpy)_2_^2+^, and both ends of probe 2 are labeled with quencher AF, and their exposed parts are all modified with azobenzene molecules which can be reversibly switched under different wavelengths of light. When the azobenzene molecule is in trans isomerization, the miRNA hybridizes with the DNA nippers, and the ECL is excited to emit light. Under ultraviolet light irradiation, azobenzene is converted to cis isomer, which inhibits DNA hybridization and turns off the ECL signal. This strategy realizes the single‐step rapid and ultrasensitive detection of miRNA, which can perform reversible operations without producing waste molecules, and maintain the DNA machine structure to extend the use time of the DNA nanomachine.

Liu and coworkers^[^ ^115^ ^]^ designed a self‐powered light‐controlled DNA walker for miRNA imaging. As shown in Figure 10C, the FAM‐labeled substrate strand and the biped DNAzyme walker are fixed on the Au nanoparticles. where the bipedal walker is composed of DNAzyme and its complementary strand. In the absence of light, the FAM fluorescence on the substrate chain is quenched. After the DNA walker is internalized into the cell, the miRNA‐21 in the cell hybridizes with the blocker strand to release the DNAzyme. The subsequent UV light induces the cleavage of photosensitive molecules, cutting off the fixed legs of DNAzyme. Subsequently, the DNAzyme cuts the substrate strand with the help of Mn^2+^, and releases the FAM‐labeled fragments to produce fluorescence. Subsequently, the DNAzyme continues to bind to the next substrate strand to carry out the next cycle to achieve the purpose of signal amplification.

In addition to intracellular RNA imaging, light responsive nucleic acid systems can also be used for DNA base variation sensing. He and colleagues^[^ ^116^ ^]^ designed and synthesized a new DNA structural unit d^Tet^U. As shown in Figure 10D, the aminopropynyl linker is connected to the uridine of the DNA structural unit, and the benzene ring with tetrazole and allyloxy group is connected through the linker. Under 302 nm light irradiation, it can induce intramolecular tetrazole‐alkene cycloaddition to produce fluorescence. The fluorescence of d^Tet^U shows different emissions in double‐stranded and single‐stranded oligonucleotides. And it will be quenched in fully complementary paired double strands. This strategy can perform mutation base detection without the addition of enzymes or other fluorescent substances.

In addition, Xiao et al.^[^ ^50^ ^]^ developed a photocage deoxyribonuclease that is simultaneously driven by UV light and Vis light, which can be synthesized by phosphorothioate chemistry. As shown in Figure 10E, two photocleavage molecules, 2‐(2‐nitrobenzyl)oxyphenyl (NBOP) and 7‐diethylaminocoumain (DEACM), are used for photocaged DNAzymes. In biological imaging performed in live cells or in vitro, the RNA cleavage activity of two light‐shrouded DNases can be selectively activated in a sequential manner under light irradiation. It lays the foundation for the logical gating of biosensing through DNAzyme.

### 4.3 Optogenetics

Optogenetics is a tool for controlling the tissue or cell activities through light. Enzymes and genetic materials are essential materials that control life activities.^[^ ^23^ ^]^ To achieve light control of biological behaviors, it is necessary to introduce photosensitivity molecules to control the function of enzymes or genetic materials, so that the behavior of organisms can be controlled optically.^[^ ^117^ ^]^ Tang et al.^[^ ^118^ ^]^ immobilized azobenzene molecules on the G‐quadruplex plane of the human telomere G4 sequence, enabling real‐time and reversible control of the folding and unfolding of G4. This controllable and sensitive way of G4 structure manipulation will be instructive for G4‐related cell senescence intervention.

**FIGURE 11:**
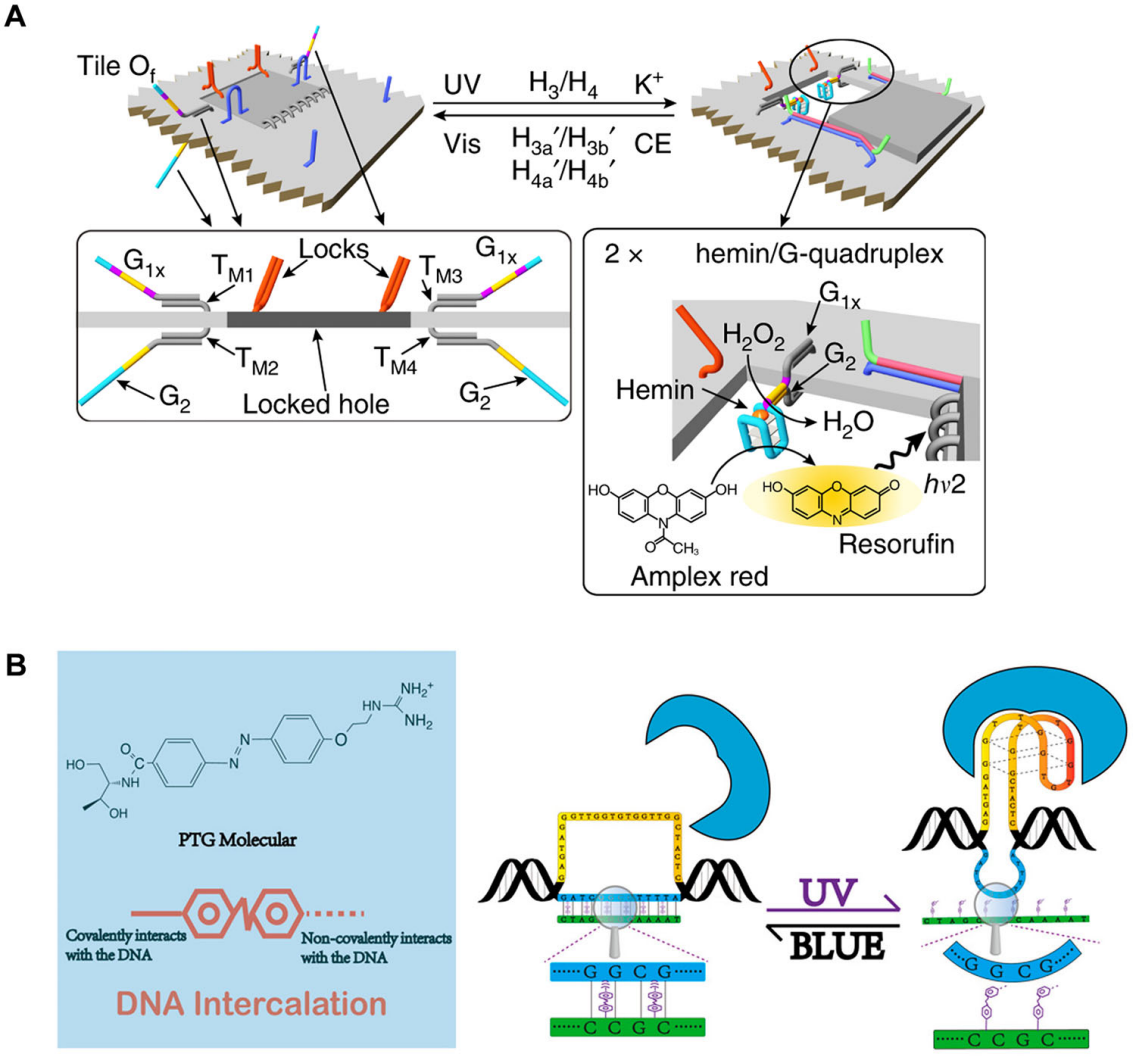
(A) The light‐induced reversible activation mechanism of hemin/G‐quadruplex deoxyribonuclease in the photogenic cavity related to origami. Reproduced with permission.^[^ ^119^ ^]^ Copyright 2019, Nature Publishing Group. (B) Schematic illustration of the light‐triggered allosteric nanoswitch. Reproduced with permission.^[^ ^120^ ^]^ Copyright 2019, American Chemical Society

Wang and colleagues^[^ ^119^ ^]^ used azobenzene‐modified oligonucleotides as light trigger units to realize reversible unlocking and locking of nanopores in DNA origami scaffolds. As shown in Figure 11A, the upper and lower surfaces of the origami are functionalized with guanosine‐rich chains G1x and G2. The “window” is locked to the origami by forming two double interlocks between the protruding strands L3/L3' and L4/L4'. And the trans‐azobenzene embedded in base pairs can stabilize the locked duplex. With the assistance of potassium ions and auxiliary hairpins, the “window” is photochemically unlocked, causing the G1x and G2 on both side of the origami to combine with each other to produce two G‐quadruplex units. The combination of heme and G‐quadruplex yields a heme/G‐quadruplex DNAzyme. The switchable “on”/“off” catalytic activity of DNAzyme is activated by sequentially triggered “mechanical” opening and closing of cavity formation and separation.

Wang and coworkers^[^ ^120^ ^]^ developed an allosteric DNA nanoswitch that controls thrombin activity through changes in the structure of two DNA hairpins. As shown in Figure 11B, the nanoswitch is mainly composed of two tail‐to‐tail DNA hairpins. The first DNA hairpin contains an aptamer sequence, and it can bind to thrombin when it has a stem‐loop structure. The second unpaired partial DNA hairpin can bind to inhibitory sequence modified with a photosensitive azobenzene derivative. By switching between UV and blue light conditions, the second DNA hairpin “hybridizes/dehybridizes” with the inhibitory sequence, so that the first DNA hairpin “cannot/can” maintain the stem‐loop structure and therefore “lost/recover” the ability to combine with the target. With these technologies, it is very possible to use antisense strategies to light‐regulate gene expression in vitro or in vivo.

### 4.4 Gene editing

Gene editing can accurately modify genes in an organism's genome through genetic engineering technology. The CRISPR‐Cas system is a widely used gene editing tool, but there are often some off‐target effects, as well as genotoxicity, translocation, and even malignant tumors.^[^ ^121^ ^]^ Therefore, controlling the CRISPR‐Cas system with high spatiotemporal resolution is beneficial for reducing off‐target effects, improving its specificity, and reducing DNA damage.

**FIGURE 12:**
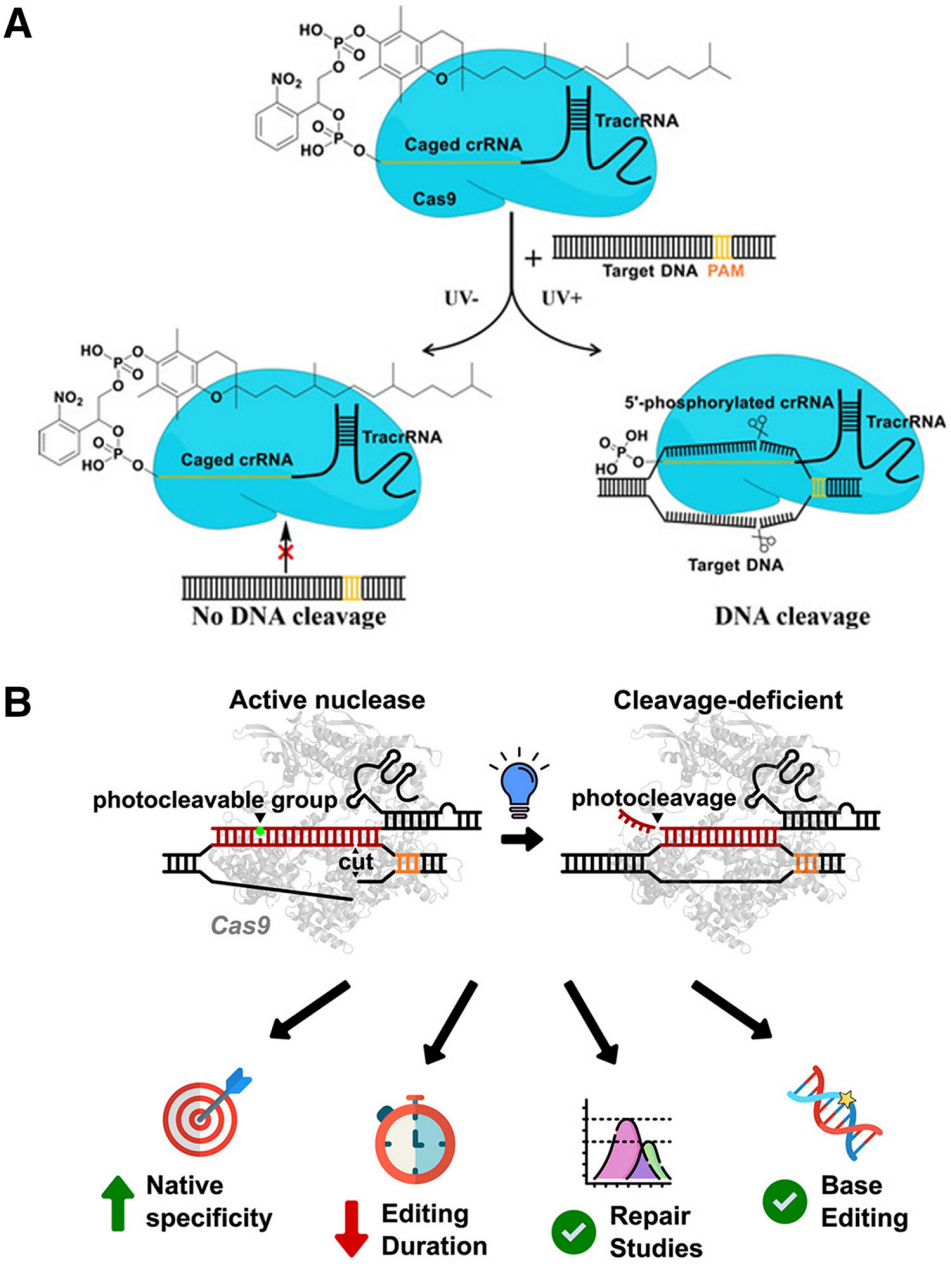
(A) Schematic diagram of light regulation of crRNA. Reproduced with permission.^[^ ^122^ ^]^ Copyright 2019, John Wiley & Sons. (B) Schematic of Cas9 deactivation mechanism. Reproduced with permission.^[^ ^123^ ^]^ Copyright 2021, Cell Press

Zhang et al.^[^ ^122^ ^]^ inactivated the CRISPR/Cas9 system by introducing vitamin E (VE)‐modified light‐labile adapter to the 5′ end of CRISPR RNA (crRNA). As shown in Figure 12A, when VE is on crRNA, the gene editing function of CRISPR‐Cas9 is effectively blocked. Under 365 nm ultraviolet irradiation, VE and photolabile linkers are removed, and the guide RNA is recovered, effectively restoring the function of the CRISPR‐Cas9 system.

In addition, Zou and colleagues^[^ ^123^ ^]^ designed a photo‐cleavable guide RNA (pcRNA) that gave the Cas9 nuclease and base editor a built‐in light‐based inactivation mechanism. As shown in Figure 12B, the photocleavable pcRNA obtained by introducing photocleavage molecules into the guide RNA can almost completely inactivate Cas9 inactivation in 1 min under light. And pcRNA can also reduce the off‐target genome editing. This pcRNA system extends the CRISPR toolbox and can be used for DNA damage repair and precise gene editing.

### 4.5 Other applications for therapeutic or diagnostic purposes

**FIGURE 13:**
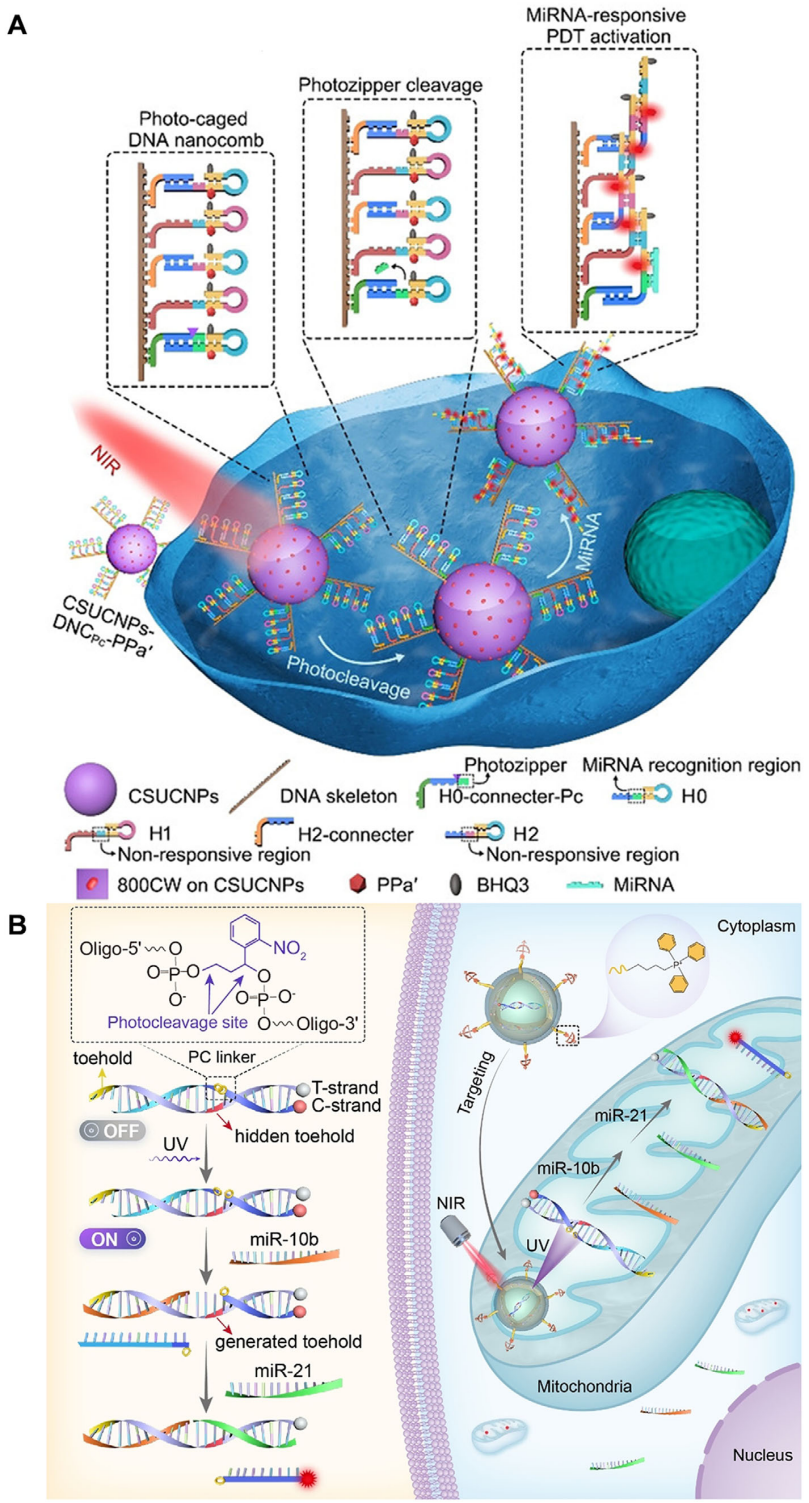
(A) Photoresponsive nucleic acid nanostructures synergize with photodynamic therapy for early‐stage cancer. Reproduced with permission.^[^ ^124^ ^]^ Copyright 2020, John Wiley & Sons. (B) Optical strand displacement reaction for mitochondrial miRNA imaging. Reproduced with permission.^[^ ^125^ ^]^ Copyright 2021, John Wiley & Sons

In addition to drug delivery or bioimaging for disease treatment or diagnosis, light responsive nucleic acid nanostructures can also be used in disease treatment or diagnosis in conjunction with other therapies through unique light‐responsive structural changes. For example, Zhang and colleagues^[^ ^124^ ^]^ designed a near‐infrared light‐driven nucleic acid nanostructure for early‐stage cancer treatment. As shown in Figure 13A, this nanostructure consists of DNA nanocombs and upconverting nanoparticles embedded with photocleavage molecules and photosensitizer molecules (ppa'). Under the illumination of 808 nm, the upconverting nanoparticles emit UV light to cleave the photocleavage molecules, exposing the miRNA recognition domain, and inducing a cascade hybridization reaction through the miRNA, thereby exposing the photosensitizer molecules. Under the excitation of blue light emitted by the upconversion nanoparticles, the photosensitizer molecules generate a large amount of reactive oxygen species, which can effectively inhibit cell proliferation and early tumor growth.

In addition, Zhao and colleagues^[^ ^125^ ^]^ reported a design to study mitotic‐related physiological events via a light‐controlled strand displacement reaction. As shown in Figure 13B, the nanoreactor consists of upconverting nanoparticles and DNA sensing probes. Under NIR illumination, the photosensitive molecule is cleaved, exposing the recognition domain of miRNA‐10b. The strand displacement reaction in miRNA‐10b exposes the recognition domain of miRNA‐21 and induces another strand displacement reaction for mitotic imaging. This method enables miRNA imaging at the subcellular level and has important implications for studying cancer‐associated mitochondria.

## 5 CHALLENGES AND PROSPECS

Light responsive nucleic acid materials are a class of outstanding functional materials for biomedical applications. One of the significant challenges is integrating photoresponsive molecules into nucleic acid sequences. Loading light‐responsive molecules onto nucleic acid sequences in a gentle manner is the key to control the structure and function changes of nucleic acid sequences. By using cyanoethyl phosphoramidite chemistry on an automatic DNA solid‐phase synthesizer, photoresponsive molecules can be incorporated into the nucleic acid strand backbone. But for other sites in the nucleic acid structure, the performance of DNA solid‐phase synthesis is mediocre. It requires researchers to continue to explore gentle methods for merging nucleic acid sequences with photoresponsive molecules. For example, N‐hydroxysuccinimide (NHS)‐mediated amino chemistry^[^ ^5^ ^]^ and click chemistry between alkynes and azides.^[^ ^126^ ^]^

Second, as the binding sites of light‐responsive molecules and nucleic acids are different, the resulting nucleic acid regulation effects are also different. The binding sites can induce various nucleic acid structure changes, such as hairpin adjustment, duplex hybridization and dehybridization strand breaks and deformations, and binding affinity changes with other biomolecules.^[^ ^52^ ^]^ It is expected that each of these methods will have more specialized applications in the future. In addition to the six binding sites mentioned above, more interesting binding sites are also hotspots worthy of further study. Velema et al. used the 2′‐OH of RNA as the binding site to introduce light‐responsive groups to optically control the catalytic function of RNA.^[^ ^127^ ^]^ Wang and coworkers^[^ ^128^ ^]^ inserted photoresponsive groups between deoxyribose and bases to regulate the hybridization of nucleic acid strands through light.

Third, effectively reducing the cytotoxicity and improving thermal stability of light‐responsive molecules is also one of the major challenges. For example, nitrosoaldehyde, a photocleavage product of o‐nitrobenzyl derivatives, can interact with surrounding proteins and is potentially toxic.^[^ ^45^ ^]^ Take the azobenzene molecule as an example, which is easily reduced under physiological conditions.^[^ ^52^ ^]^ Generally, only about 30% of the azobenzene molecules in the 35‐mer DNA duplex can be photoisomerized into the cis conformation at 37°C.^[^ ^129^ ^]^ Therefore, it is very necessary to develop efficient photoisomerization of azobenzene to anchor DNA chain to meet the light control actuation at room temperature. Kou et al.^[^ ^130^ ^]^ found that by integrating azobenzene into the DNA strand through a glycerol linker, the light‐controlled operation efficiency of the hybrid double‐strand at room temperature has been greatly improved. By modifying the cationic guanidine tail on the azobenzene molecule, Bergen and colleagues realized the reversible conversion of the isomerization of the azobenzene molecule at room temperature.^[^ ^91^ ^]^ These works will further optimize the application feasibility of the light responsive nucleic acid system. Morihiro and colleagues^[^ ^131^ ^]^ used capillary electrophoresis‐SELEX technology to optimize the azobenzene‐modified DNA aptamer and increase its affinity for thrombin in vitro. These evolved azobenzene‐modified aptamers exhibit strong thrombin affinity and high photoisomerization efficiency.

In addition, for photoresponsive systems, the phototoxicity and tissue penetration of excitation light are unavoidable problems. Generally speaking, most photoresponsive molecules are excited in the UV region. This UV light excitation mode inevitably produces phototoxicity to tissue cells.^[^ ^85^ ^]^ In addition, the scattering and absorption of UV light by the skin results in poor penetration of UV light, while the absorption of light by hemoglobin and water results in an ideal irradiation window of 650–900 nm.^[^ ^132^ ^]^ Therefore, it is particularly important to adjust the excitation light to the NIR light region. In recent years, researchers have proposed methods such as two‐photon absorption and upconversion luminescence to avoid many defects of UV light excitation.^[^ ^89^, ^133^ ^]^ Two‐photon excitation has been widely studied as an effective candidate, but conventional two‐photon excitation often uses high‐intensity amplified pulsed lasers, which limits the application of the two‐photon method. Donato et al.^[^ ^134^ ^]^ found that when the alkoxy groups in the biphenyl 2‐(o‐nitrophenyl)propyl and its derivatives are replaced by better electron‐donating groups (such as dialkylamino), the two‐photon sensitivity of photocleavage groups can be significantly enhanced and result in a two‐photon uncaging cross‐section as high as 11 GM. In addition, up‐conversion luminescence has also become an effective means to overcome the limited penetration and phototoxicity of UV light excitation.^[^ ^135^ ^]^ In the biomedical applications of nucleic acids, upconversion luminescence is usually achieved by upconversion nanoparticles (UCNPs), which convert NIR radiation into emitted light with multiple wavelengths.^[^ ^136^ ^]^ UCNPs are nanomaterials doped with rare earth ions, which can continuously absorb multiple low‐energy near‐infrared photons.^[^ ^137^ ^]^ Unlike two‐photon excitation, the upconversion luminescence can absorb photons to accumulate energy through multiple intermediate states. Therefore, it can reach a high energy level by absorbing multiple photons and then emitting photons with shorter wavelengths than a single excitation photon. In this process, a real intermediate state appears. The relatively stable intermediate state can continue to absorb energy from the photon to reach a higher excited state, and then emit ultraviolet or visible light by transitioning back to the ground state.^[^ ^33^, ^88^ ^]^ Zhao et al.^[^ ^133^ ^]^ designed a NIR light‐controlled DNA nanostructure, and used UCNPs to regulate the DNA hairpin structure, which was used as a converter of NIR light to emit UV light. Such nanostructure can be used to detect miRNA in vivo and in vitro with high spatiotemporal precision.

In order to further realize the practical application of light responsive nucleic acids in biomedicine, several challenges should be overcome. 1) Develop more binding methods and binding sites for light responsive molecules and nucleic acid sequences to enrich the light control mechanism. 2) The light‐activated irradiation window is transferred to the NIR light region to improve the permeability of the excitation light and reduce the phototoxicity. 3) Design and develop more photoresponsive molecules with various functional groups.

## 6 CONCLUSION

Light responsive nucleic acid is an outstanding biological material, which is widely used in biomedical applications. The structure, shape, and hybridization/dehybridization of light‐controlled nucleic acid can be reversibly or irreversibly adjusted through the adjustment of the light intensity wavelength and irradiation time. Various photoresponsive molecules can be introduced into different binding sites of nucleic acid sequences through various methods and exert various effects. This makes light responsive nucleic acids have great potential in drug delivery, biosensing, optogenetics, and gene editing.^[^ ^44^, ^98^, ^102^, ^106^, ^138^ ^]^

However, most of the reported light responsive nucleic acid nanosystems are only at the stage of theoretical research. There are still very few researches of light responsive nucleic acid materials for in vivo studies or preclinical studies. In the future, there should be more reports on in vivo or preclinical studies to strengthen the application of light responsive nucleic acid nanosystems. Although there are still obstacles to the realization of light responsive nucleic acid nanosystems in biomedical clinical applications. With the continuous advancement of the regulation mechanism and the continuous improvement of performance, light responsive nucleic acid nanosystems will show greater potential in biomedical applications.

## CONFLICT OF INTEREST

The authors declare no conflict of interest.
